# The Efficiency and Cost‐Effectiveness of 3D‐Printed Patient‐Specific Guide Plate for Patients Undergoing Open‐Wedge High Tibial Osteotomy: A Multicentered Randomized Controlled Trial

**DOI:** 10.1111/os.70259

**Published:** 2026-02-15

**Authors:** Runhua Zhou, Yanjie Guo, Ruixin Wang, Manrong Xu, Qinglin Kang, Yun Shen, Jia Xu, Da Zhong, Shengdi Lu

**Affiliations:** ^1^ Department of Orthopedics Shanghai Sixth People's Hospital Affiliated to Shanghai Jiao Tong University School of Medicine Shanghai China; ^2^ School of Public Health Fudan University Shanghai China; ^3^ Department of Endocrinology Shanghai Sixth People's Hospital Affiliated to Shanghai Jiao Tong University School of Medicine Shanghai China; ^4^ Pennington Biomedical Research Center Baton Rouge Louisiana USA; ^5^ Department of Orthopaedics, Xiangya Hospital Central South University Changsha China

**Keywords:** cost–benefit analysis, osteoarthritis, osteotomy, patient‐specific instrumentation, randomized controlled trial

## Abstract

**Purpose:**

Open‐wedge high tibial osteotomy (OWHTO) is established for young, active patients with medial knee osteoarthritis. Patient‐specific 3D‐printed guide plates have been introduced to improve surgical precision and efficiency, but evidence of clinical and economic benefit is limited. We aimed to determine whether a 3D‐printed patient‐specific guide plate improves efficiency, functional outcomes, and cost‐effectiveness compared to standard OWHTO.

**Methods:**

In this multicenter randomized trial, patients scheduled for OWHTO were allocated to either conventional planning (control) or surgery using a patient‐specific 3D‐printed guide plate between November 2020 and June 2024. The primary endpoint was the 12‐month Western Ontario and McMaster Universities Osteoarthritis Index (WOMAC) pain score. Secondary outcomes included knee range of motion (flexion in degrees), 30‐s chair‐stand test (number of stands), operative time, and health economic measures (direct costs and quality‐adjusted life years). Analyses were by intention‐to‐treat using appropriate statistical tests.

**Results:**

A total of 180 patients (mean age 55 years, 56.7% male) were randomized equally between groups. At 12 months, mean WOMAC pain was 15.2 (SD 8.4) in the guide‐plate group and 15.6 (SD 8.7) in controls, with no significant difference (*p* = 0.74). The guide‐plate group showed significantly greater knee flexion (mean 128° vs. 122°; *p* = 0.04) and a higher 30‐s chair‐stand count (14.2 vs. 12.5 stands; *p* = 0.02) than controls. There were no other significant between‐group differences in clinical scores. Mean total cost per patient was not statistically significant in the ITT analysis (*p* = 0.094). Quality‐adjusted life years did not differ between groups, yielding no cost‐effectiveness advantage. These findings echo prior reports that OWHTO techniques with higher costs have similar patient outcomes.

**Conclusion:**

Using a 3D‐printed patient‐specific guide plate did not improve the primary pain outcome or overall functional outcome compared to standard OWHTO. It yielded minor gains in knee flexion and chair‐stand performance, but at greater cost. No overall cost‐effectiveness benefit was observed. Routine use of this technology for OWHTO is not supported by our findings.

**Level of Evidence:**

Level I, randomized controlled trial.

**Trial Registration:**

Chinese Clinical Trial Registry (https://www.chictr.org.cn/): ChiCTR2000038619

## Graphical abstract

In a multicenter randomized trial of open‐wedge high tibial osteotomy, use of a 3D‐printed patient‐specific guide plate noted no adjusted difference in 12‐month WOMAC pain; small gains in ROM/chair‐stand; higher device cost; no QALY gain; no overall cost‐effectiveness.

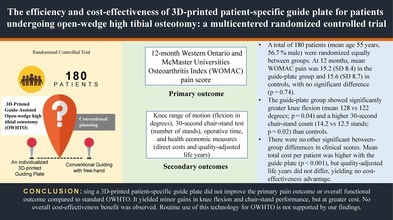

**Abbreviations**
3D: three‐dimensional
AP: anteroposterior (as in AP radiograph)
BMI: body mass index
CI: confidence interval
CNY: Chinese Yuan (currency)
CONSORT: Consolidated Standards of Reporting Trials
CT: computed tomography
EQ‐5D‐5L: EuroQol 5‐Dimension 5‐Level (quality‐of‐life questionnaire)
HTO: high tibial osteotomy
ICER: incremental cost‐effectiveness ratio
IKDC: International Knee Documentation Committee
ITT: intention‐to‐treat
KL: Kellgren–Lawrence (grading scale for osteoarthritis)
KOA: knee osteoarthritis
MAD: mechanical axis deviation
MCID: minimally clinically important difference
MCL: medial collateral ligament
OA: osteoarthritis
OWHTO: open‐wedge high tibial osteotomy
PP: per‐protocol
PSI: patient‐specific instrumentation
ROM: range of motion
SD: standard deviation
WBL: weight‐bearing line
WOMAC: Western Ontario and McMaster Universities Osteoarthritis Index

## 1 Background

High tibial osteotomy (HTO) is an established joint‐preserving procedure for younger, active patients with medial compartment knee osteoarthritis and varus malalignment [1, 2, 3, 4]. Medial open‐wedge HTO (OWHTO) shifts the weight‐bearing axis laterally, offloading the diseased medial compartment and alleviating pain, often yielding excellent long‐term outcomes in appropriately selected patients. Studies report that OWHTO can significantly improve knee function and even enable over 90% of young patients to return to work or sports within a year of surgery [3, 4]. Durable benefits have been documented at 5–10 years postoperatively, with maintained joint function and high patient satisfaction [3, 4]. These results underscore the value of HTO as a cost‐effective alternative to knee arthroplasty in the younger osteoarthritic population.

Despite its success, OWHTO is a technically demanding operation. Achieving the planned correction with freehand techniques can be challenging, since the surgeon must precisely create and distract the bony wedge based on preoperative planning and intraoperative fluoroscopic guidance [5]. Even minor deviations in the sawing orientation or opening height can lead to suboptimal alignment or unintended changes in posterior tibial slope, which may compromise long‐term outcomes. In practice, conventional methods relying on 2D long‐leg radiographs and repeated fluoroscopy often yield inconsistent accuracy in alignment correction [5, 6, 7]. A systematic review found that many patients do not achieve the exact planned mechanical axis using standard techniques, and a meta‐analysis confirmed that computer‐navigated HTO offers statistically more precise alignment than the freehand approach [5, 6]. However, navigation systems are costly and have shown only modest improvements over conventional fluoroscopic guidance in clinical outcomes. Furthermore, the need for extensive intraoperative imaging with freehand OWHTO prolongs operative time and exposes patients and surgical staff to considerable radiation. These limitations of the traditional technique, including reliance on surgeon experience, variability in accuracy, risk of hinge fracture or loss of correction, and increased radiation exposure, highlight the need for tools that can make OWHTO more reliable and reproducible.

In recent years, advances in three‐dimensional (3D) imaging and printing technology have led to the development of patient‐specific instrumentation (PSI) for OWHTO [7, 8, 9, 10, 11, 12]. Using the patient's own imaging data (a preoperative CT scan), a 3D virtual plan of the osteotomy can be created and a custom cutting guide can be 3D‐printed to fit the patient's bone anatomy. The goal of these patient‐specific guides is to translate the preoperative plan to the operating room with high precision, thereby standardizing the osteotomy cut and wedge opening according to the planned alignment. Early clinical reports have been encouraging: in a pilot series of PSI‐guided OWHTO, Yang et al. [8] achieved the planned post‐operative weight‐bearing line (WBL) while maintaining the tibial slope within 0.2° of preoperative value. They noted that the PSI technique was “time‐saving, radiation‐reducing, and relatively easy to use,” with all patients experiencing improved range of motion and pain relief by 3 months post‐surgery [8]. Similarly, Mao et al. [11] reported in a prospective comparative study that patient‐specific 3D cutting guides significantly improved the accuracy of alignment correction compared to conventional freehand HTO. Notably, patients treated with PSI guides showed better early functional scores (IKDC and Lysholm) in the weeks after surgery, reflecting faster recovery. Collectively, these studies suggest that 3D‐printed patient‐specific guides can enhance the precision of OWHTO and potentially improve short‐term patient outcomes by facilitating more accurate and efficient surgery [10, 11, 12].

However, it remains unclear whether the improved surgical accuracy and perioperative efficiency associated with PSI translate into superior long‐term clinical benefits and justified costs. Designing and manufacturing patient‐specific guides incur additional preoperative time and expense, and robust evidence on their cost‐effectiveness is still lacking [13]. To address this gap, we conducted a multi‐center randomized controlled trial comparing the 3D‐printed patient‐specific guide plate technique against the conventional freehand technique for medial OWHTO. The primary aim of this trial was to evaluate whether using a patient‐specific 3D guide improves pain outcomes (primary outcome: WOMAC pain score) while achieving more accurate leg alignment. Secondary aims included assessing knee function (range of motion, muscle strength, WOMAC function/stiffness, Lysholm score), mechanical axis deviation correction, patient quality of life, and a comprehensive analysis of economic outcomes, as well as any adverse events. We hypothesized that the PSI‐guided OWHTO would lead to superior early pain relief and functional recovery, with more consistent restoration of the mechanical axis, compared to conventional planning and fluoroscopic guidance. Moreover, we anticipate that greater surgical precision and efficiency will translate into improved cost‐effectiveness, validating the use of 3D‐printed patient‐specific guides in routine HTO practice.

## 2 Methods

### 2.1 Study Design and Setting

This multicenter randomized controlled trial used a parallel group design and was pre‐registered at the Chinese Clinical Trial Registry. Recruitment occurred between November 2020 and June 2024 at two tertiary A hospitals in China. Participants and outcome assessors were blinded to group assignment; surgeons were not blinded. Randomization and allocation concealment are described in subsequent sections of the protocol.

### 2.2 Ethical Statement

The trial was approved by the Ethics Committee. All study procedures were conducted in accordance with the Declaration of Helsinki of 1964 and its later amendments. Written informed consent was obtained from all participants prior to enrollment, and participants were informed of their right to withdraw from the study at any time. All personal data were anonymized (de‐identified) to ensure participant privacy and confidentiality; therefore, specific consent to publish such data was not required. No financial or other compensation was provided to participants for their participation in this study.

### 2.3 Participants

All participants were screened from the waiting list of OWHTO from the outpatient clinic of two participating centers. Patients meeting standard indications for OWHTO were contacted on the same day of admission and were consecutively recruited if informed consent was signed.

Inclusion criteria were symptomatic, isolated medial compartment knee osteoarthritis of Kellgren–Lawrence grade II–III with a varus alignment of 5°–15° on weight‐bearing full‐leg radiographs. Additional requirements included age 18–55 years, a physically active lifestyle, intact knee range of motion, and sufficient cognitive and functional capacity for postoperative rehabilitation.

Key exclusion criteria included any prior surgery on the affected knee (such as a previous osteotomy, ligament reconstruction, or meniscectomy), the presence of any inflammatory arthritis (e.g., rheumatoid arthritis) or advanced osteoarthritis in another compartment (Kellgren–Lawrence grade IV in the lateral or patellofemoral compartment), significant bone or vascular pathology resulting in poor bone stock (e.g., advanced osteoporosis or major vascular disease), a fixed flexion contracture greater than 10° or varus deformity greater than 15°, moderate‐to‐severe patellofemoral osteoarthritis (greater than grade II), and any neurologic or psychiatric condition that could impair participation or follow‐up. Body mass index was recorded for all patients but was not used as an exclusion criterion. The full eligibility criteria are summarized in Table 1.

**TABLE 1:** Eligible criteria.

Inclusion criteria
Adults aged 18–55 years with symptomatic isolated medial compartment knee osteoarthritis (Kellgren–Lawrence grade II–III) and varus knee alignment of 5°–15° (suitable for open‐wedge high tibial osteotomy) Physically active (non‐sedentary) lifestyle, with ability to comply with the postoperative rehabilitation program Intact knee range of motion, with adequate cognitive and functional capacity to participate in rehabilitation

Exclusion criteria
Any prior surgery on the affected knee (including previous osteotomy, ligament reconstruction, or meniscectomy) Any inflammatory arthritis (such as rheumatoid arthritis) or advanced osteoarthritis in another compartment (Kellgren–Lawrence grade IV in the lateral or patellofemoral compartment) Severe bone or vascular pathology leading to poor bone stock (such as advanced osteoporosis or major vascular disease) Excessive knee deformity (varus > 15°) or a fixed flexion contracture > 10° Moderate‐to‐severe patellofemoral osteoarthritis (greater than grade II) Any neurologic or psychiatric condition that could impede postoperative rehabilitation or follow‐up compliance

### 2.4 Intervention

#### 2.4.1 Design and Validation of 3D‐Printed Patient‐Specific Guide Plate

The detailed description of the design and validation of the 3D‐Printed Patient‐Specific Guide Plate is shown in Supporting Information.

#### 2.4.2 Surgical Procedure

Five surgeons with over 5 years of OWHTO experience from two participating hospitals were involved in this study. All surgeons adhered to a standardized operative protocol to ensure a consistent technique across centers. Key surgical steps were uniform for all cases. Both centers employed the same preoperative planning method and patient positioning; the osteotomy was performed with an identical approach and angle, and all osteotomies were fixed using the same type of locking plate without any bone graft. This protocol was established collaboratively before the trial, and all surgeons followed it strictly to minimize inter‐center variability in surgical technique.

For each patient in the guide‐plate group, we employed a standardized 3D design and printing workflow (full technical details are provided in the S1). Briefly, the patient's preoperative CT scans were segmented to construct a 3D digital model of the proximal tibia. Using this model, we performed computer‐assisted preoperative planning to determine the required osteotomy correction (targeting the weight‐bearing line at Fujisawa's point on the tibial plateau) and to design a patient‐specific cutting guide. The guide was custom‐modeled to fit the anteromedial surface of the tibia, aligning with key bony landmarks and incorporating an internal “bursa interface” for soft‐tissue clearance, and it featured built‐in slots to guide the drilling and sawing during the osteotomy. The finalized guide design was exported as an STL file and fabricated with a 3D printer using a biocompatible photosensitive resin. Each guide's fit and the planned osteotomy trajectory were virtually validated by overlaying the guide on the patient's 3D bone model prior to printing. No separate physical test‐fitting on a bone model was performed. After printing, the guide was sterilized (via standard high‐temperature and pressure autoclaving) and prepared for intraoperative use. Intraoperatively, a standard anteromedial approach exposed the proximal tibia (with MCL and hamstring retracted). The patient‐specific guide was applied and temporarily fixed in place (usually with Kirschner wires); a guide K‐wire was drilled at the planned osteotomy level toward the fibular head until a preset depth (the planned hinge point) was reached. A “stop” wire (often called a golden guide wire) was used to protect the lateral cortex. The osteotomy was then performed with an oscillating saw through the guide's slot until the bone cut was completed [1, 8, 14]. The osteotomy gap was gradually opened with laminar spreaders to achieve the target correction (shifting the weight‐bearing line to Fujisawa's point), which was determined by the preoperative plan implemented via the guide [1, 8, 14]. A precontoured TomoFix locking plate was then placed medially over the opened gap; the plate holes were aligned to predrilled proximal cortex holes, and screws were inserted sequentially (proximal and distal) to lock the plate and hold the correction [14].

The control group used the conventional freehand technique with radiographic planning. Preoperatively, standing full‐length (hip‐to‐ankle) AP radiographs were obtained and used to measure the mechanical axis and determine the required wedge opening (typically targeting a slight valgus overcorrection). In surgery, similar exposure was achieved; then one or two K‐wires were drilled from the medial tibia toward the lateral cortex at the planned angle (aiming at the fibular head). These guide wires defined the saw cut direction. Under intraoperative fluoroscopic imaging, the osteotomy was performed freehand using an oscillating saw along the K‐wire guide, preserving a lateral hinge. The osteotomy was opened with an osteotome/laminar spreader to the preplanned height, verifying alignment and hinge integrity on x‐ray. The TomoFix plate was then applied and fixed with locking screws in the same manner as above. In both groups, the opening gap was not filled with any bone graft or substitute; stable fixation with the locking plate alone was relied on for healing. The detailed surgical procedure was shown in Supporting Information.

#### 2.4.3 Postoperative Care and Rehabilitation Protocol

Postoperative care including pain management, antibiotic prophylaxis, thromboprophylaxis, elevation and mobilization. The detailed postoperative care protocol is provided in the Supporting Information.

Patients in both centers will undergo a standardized 12‐week outpatient physiotherapy program. Sessions are supervised four times per week during weeks 1–4, three times per week in weeks 5–8, and twice per week in weeks 9–12. Each week, therapists assess joint range of motion, muscle strength, gait, and pain to guide progression. Early‐phase goals focus on pain and swelling control, achieving full knee extension, and gradually increasing flexion (target 90° by week 4). Later goals include quadriceps and hip strengthening, normalizing gait, and functional tasks. Detailed week‐by‐week interventions and progression criteria are provided in the Supporting Information.

### 2.5 Outcome Measures

#### 2.5.1 Primary and Secondary Outcome

The primary outcome was the Western Ontario and McMaster Universities Osteoarthritis Index (WOMAC) pain subscale (0–100 point scale, higher scores indicating worse pain) [15]. Secondary clinical outcomes included radiographic alignment, knee mobility, muscle strength, functional tests, patient‐reported scores, and safety measures. Mechanical axis deviation (MAD) of the lower limb was measured on weight‐bearing full‐leg radiographs as the perpendicular distance (mm) from the hip–ankle mechanical axis line to the center of the knee joint (a positive medial or lateral offset indicating varus or valgus malalignment, respectively) [16]. Active knee range of motion (flexion–extension in degrees) was assessed with a standard goniometer, a reliable and valid method for quantifying joint mobility in knee disorders [17]. Isometric knee flexion strength (hamstring muscle strength) was measured as the peak voluntary contraction torque at a fixed knee angle (60° flexion) using an isokinetic dynamometer, and dynamic knee flexion strength was measured as the peak concentric knee flexion torque at a predefined angular velocity, both normalized to the contralateral (unaffected) limb as a percentage to account for inter‐individual differences [18]. Lower‐limb functional performance was evaluated with the 30‐s chair sit‐to‐stand test, in which participants, seated in a standard armless chair (seat height 43 cm) with arms folded across the chest, stand up fully and sit down as many times as possible within 30 s; the total number of correct stands was recorded as the score [19].

Patient‐reported secondary outcomes included the WOMAC physical function and stiffness subscales (each scored 0–100, higher = worse) in addition to the pain subscale. We also administered the Lysholm Knee Score (0–100, higher scores indicating better knee function) to evaluate knee symptoms and functional disability [20]. Health‐related quality of life was measured using the EuroQol 5‐Dimension 5‐Level (EQ‐5D‐5L) questionnaire, and responses were converted into a utility index (where 1 represents perfect health and 0 represents death) using the established EQ‐5D‐5L value set [21].

#### 2.5.2 Cost Measures

We collected comprehensive cost outcomes to enable an economic evaluation from a societal perspective. All relevant costs associated with the intervention and its outcomes were captured, including direct medical costs (costs of postoperative rehabilitation sessions, physical therapy, follow‐up clinic visits, and medications) and direct non‐medical costs related to care (patient transportation expenses for medical visits and any specialized nutritional supplements during recovery). Indirect costs due to productivity loss were also recorded, encompassing lost wages or income for patients during recovery or temporary disability, as well as any lost income for family members or caregivers who needed to provide assistance [22]. Cost data were obtained from hospital billing records for medical services and from patient self‐reports for non‐reimbursed expenses such as transportation and wage losses. All costs are reported in 2024 Chinese Yuan (CNY), as 2024 was the end of the enrollment period. Costs incurred in earlier years were adjusted to 2024 price levels using the national consumer price index to account for inflation. Consistent with recommended practices in health economic evaluations, costs and outcomes occurring beyond the first year postoperative were discounted at an annual rate of 3% to reflect time preference. This approach to costing, including direct medical, direct non‐medical, and indirect costs, aligns with a broad societal viewpoint for economic analysis and with established guidelines for conducting cost‐effectiveness studies. All outcome and cost data were recorded using pre‐specified case report forms by trained research staff, ensuring standardized data collection across participants.

### 2.6 Adherence, Adverse Events, and Serious Adverse Events

Adherence to the postoperative rehabilitation program was tracked throughout the study: attendance at prescribed physiotherapy sessions was recorded, an objective indicator of rehabilitation adherence [23]. All adverse events (AEs) and serious adverse events (SAEs) were monitored and documented from the time of surgery until final follow‐up [24]. AEs were defined as any untoward medical occurrences affecting participants, and SAEs were defined according to standard criteria (any event that results in death, is life‐threatening, requires or prolongs hospitalization, or causes significant disability). Any such events were reviewed by the study safety committee and managed according to trial protocol.

### 2.7 Data Collection

All outcome measures were collected at 3‐, 6‐, and 12‐month post‐operation by assessors blinded to group allocation.

### 2.8 Sample Size Calculation

The planned sample size was determined based on the WOMAC pain subscale (0–100 scale) as the primary outcome in accordance with CONSORT guidelines. We chose a minimally clinically important difference (MCID) informed by the literature: Taslakian et al. [25] defined the WOMAC pain MCID as a 4‐point change on the 0–20 scale (equivalent to 20 points on the 0–100 scale). Others have suggested that a change of roughly 10–20 points (10%–20%) represents a minimal meaningful improvement [26]. Conservatively, we assumed an MCID of about 12 points (12%) on the 0–100 WOMAC pain scale. The standard deviation (SD) of baseline WOMAC pain was estimated from prior studies: in a large cohort of knee OA patients the mean baseline score was 26.5 (SD = 19), so we used SD = 20. Using a standard deviation of 20 points, 80% power, and *α* = 0.05, we estimated that 44 patients per group are required to detect a 12‐point difference. To ensure robustness, we conducted sensitivity analyses for smaller MCIDs of 10 points and 8 points, which indicated sample size requirements of 63 and 98 patients per group, respectively (details in Supplementary Methods). Our planned enrollment (*n* = 90 per group, allowing 25% dropout) provides adequate power to detect even an 8‐point MCID. This sample size was deemed sufficient given the effect size estimates in knee osteoarthritis literature and in high tibial osteotomy populations.

### 2.9 Randomization, Blinding, and Allocation

After enrollment, participants were randomly assigned in a 1:1 ratio to either the 3D guide‐plate group or the standard surgery (control) group. The randomization sequence was computer‐generated by an independent statistician *not* involved in patient enrollment or assessment. To ensure balanced group sizes while preventing prediction of assignments, permuted blocks of random sizes (block size 6) were used in the sequence generation. Allocation concealment was achieved using sequentially numbered, opaque, sealed envelopes prepared ahead of time by an independent research assistant. These envelopes were opened only after a patient had been formally enrolled (post‐consent), thereby preventing any foreknowledge of the upcoming group assignment by the investigators. At each center, the site investigators (surgeons and research coordinators) enrolled the patients and then assigned them to the allocated intervention by opening the next sealed envelope in the sequence. This implementation ensured that the person determining each patient's group had no access to the assignment sequence in advance, minimizing the risk of selection bias in group allocation.

Blinding was maintained through a single‐blind design. While operating surgeons could not be blinded to the surgical technique, all patients and outcome assessors remained unaware of each participant's group assignment. Each trial arm was managed in a separate care pathway with independent clinical teams, so that patients in one group had no contact with staff or patients from the other group's treatment arm. This separation, along with standardizing postoperative care, prevented patients from inadvertently learning about the alternate intervention and helped ensure identical clinical management apart from the surgical technique itself. Outcome evaluations (such as functional assessments and radiographic measurements) were performed by trained assessors who were not involved in the surgeries and who were blinded to group allocation. Participants were explicitly instructed not to disclose their assigned group to the assessors at any point. These precautions were implemented to uphold blinding and thereby minimize performance and detection bias. In summary, the randomization and allocation process was conducted rigorously, using an independent computer‐generated sequence with concealed assignments, followed by blinding of participants and evaluators to reduce potential allocation‐related biases in this trial.

### 2.10 Statistical Analysis

The detailed description of statistical analysis was shown in Supporting Information.

## 3 Results

### 3.1 Participants

**FIGURE 1:**
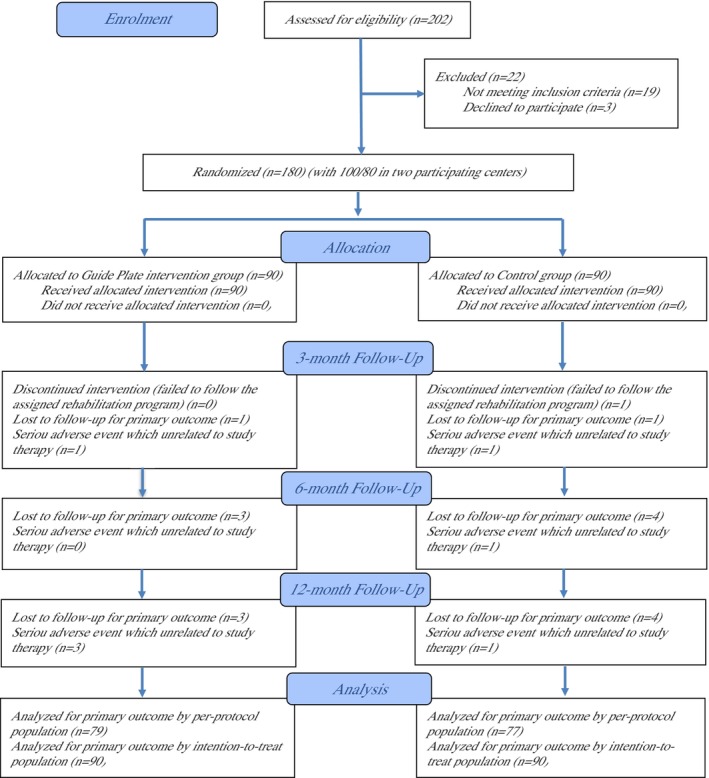
This figure shows flow diagram of the progress through the phases of a randomized trial of two groups (i.e., enrolment, intervention allocation, follow‐up, and data analysis).

The flow diagram of the study is shown in Figure 1. Baseline characteristics of the ITT population are shown in Table 2. Fifty‐three of 90 (58.89%) were male in the guide plate versus 49 of 90 (54.44%) in controls (*p* = 0.547). Mean (SD) age was 55 (2.94) versus 55 (2.80) years (*p* = 0.586) and BMI was 23 (2.81) versus 23 (2.86) kg/m^2^ (*p* = 0.856). Occupational status (manual vs. nonmanual), education level, and insurance were similar (*p* = 0.765, 1.000, 0.605). Smoking (33.33% vs. 25.56%), alcohol (18.89% vs. 21.11%), analgesic use (23.33% vs. 25.56%), living alone (34.44% vs. 33.33%), and walking aid use (64.44% vs. 67.78%) were comparable (all *p* > 0.25). Comorbidities and KOA severity (Kellgren–Lawrence grade II 75.56% vs. 74.44%; grade III 24.44% vs. 25.56%; patellofemoral grade I 72.22% vs. 73.33%; grade II 27.78% vs. 26.67%) were similar. Mean mechanical axis deviation was 30 (4.06) versus 30 (4.29) mm (*p* = 0.581). Knee function and patient‐reported outcomes (WOMAC pain, function, stiffness; Lysholm; EQ‐5D‐5L) were similar between groups (all *p* ≥ 0.646). Baseline characteristics were similar in the per‐protocol population (Table S1).

**TABLE 2:** Baseline characteristics of the guide plate group and the control group (intention‐to‐treat analysis).

ITT	Characteristics^a^	Guide plate (*N* = 90)	Control (*N* = 90)	*p*
Baseline characteristics	Male gender (no. [%])	53 (58.89)	49 (54.44)	0.547
	Age (years)	55 (2.94)	55 (2.80)	0.586
	BMI (kg/m^2^)	23 (2.81)	23 (2.86)	0.856
	Occupation (no. [%])			0.765
	*Manual worker*	48 (53.33)	46 (51.11)	
	*Non‐manual worker*	42 (46.67)	44 (48.89)	
	Education level (no. [%])			1.000
	*Lower than high school*	79 (87.78)	79 (87.78)	
	*Equal/higher to high school*	11 (12.22)	11 (12.22)	
	Insurance type (no. [%])			0.605
	*Government*	81 (90.00)	80 (88.89)	
	*Commercial*	9 (10.00)	9 (10.00)	
	*Self‐financed*	0 (0.00)	1 (1.11)	
	Current Smoker (no. [%])	30 (33.33)	23 (25.56)	0.252
	Current alcohol use (no. [%])	17 (18.89)	19 (21.11)	0.709
	Paracetamol and NSAIDs (no. [%])	21 (23.33)	23 (25.56)	0.729
	Living alone (no. [%])	31 (34.44)	30 (33.33)	0.875
	Walking aid (no. [%])	58 (64.44)	61 (67.78)	0.637
Comorbid illness	Osteoporosis (no. [%])	59 (65.56)	57 (63.33)	0.755
	Hypertension (no. [%])	8 (8.89)	6 (6.67)	0.578
	Diabetes (no. [%])	11 (12.22)	12 (13.33)	0.823
	COPD (no. [%])	7 (7.78)	6 (6.67)	0.773
	Peripheral vascular disorder (no. [%])	6 (6.67)	5 (5.56)	0.756
	Arthritis in other joints (no. [%])	14 (15.56)	13 (14.44)	0.835
KOA severity	Kellgren‐Lawrence grade (no. [%])			0.863
	*Grade II*	68 (75.56)	67 (74.44)	
	*Grade III*	22 (24.44)	23 (25.56)	
	Patellofemoral OA (no. [%])			0.867
	*Grade I*	65 (72.22)	66 (73.33)	
	*Grade II*	25 (27.78)	24 (26.67)	
	Mechanical axis deviation (MAD) (mm)	30 (4.06)	30 (4.29)	0.581
Knee function	ROM of knee flexion to extension (°)	105 (7.82)	105 (8.00)	0.707
	Isometric knee flexion strength (% of unaffected side)	83 (5.19)	82 (4.98)	0.815
	Dynamic knee flexion strength (% of unaffected side)	85 (5.50)	85 (4.63)	0.396
	30‐s chair sit‐to‐stand test (times)	11 (1.54)	11 (1.45)	0.619
PROMs	WOMAC‐pain	61 (7.30)	61 (7.11)	0.877
	WOMAC‐function	59 (3.05)	59 (3.39)	0.678
	WOMAC‐Stiffness	57 (7.93)	57 (7.96)	0.888
	Lysholm knee score	60 (5.13)	60 (5.57)	0.646
	EQ‐5D‐5L‐utility	0.35 (0.10)	0.35 (0.08)	0.806

Abbreviations: BMI, body mass index; COPD, Chronic Obstructive Pulmonary Disease; EuroQol‐5 Dimension, 5‐Level; ITT, intention‐to‐treat; KOA, knee osteoarthritis; NSAIDs, Non‐Steroidal Anti‐Inflammatory Drugs; PROMs, patient‐reported outcome measures; ROM, range of motion; WOMAC, Western Ontario and McMaster Universities Osteoarthritis Index.

^a^ Values were reported as mean (standard deviation) for age, BMI, mechanical axis deviation (MAD), all variables of knee function and PROMs, others were reported as number (percentage).

### 3.2 Primary and Secondary Outcomes

For the primary outcome (WOMAC pain), no statistically significant between‐group differences were observed after adjustment at 3, 6, or 12 months (all adjusted *p* > 0.05). However, at the 12‐month primary endpoint (the timepoint for which the trial was powered), the unadjusted analysis showed a slightly greater pain improvement in the guide‐plate group. This difference did not remain significant after adjusting for baseline WOMAC pain. Similarly, no significant between‐group differences were found at any follow‐up for mechanical axis deviation, isometric knee flexion strength, dynamic knee flexion strength, Lysholm knee score, or EQ‐5D‐5L utility (all *p* > 0.05) (Tables 3 and 4). In contrast, the guide‐plate group showed significantly greater knee range of motion (flexion‐extension) at each follow‐up. The adjusted mean difference in ROM was 2.686° (95% CI 1.120–4.251; *p* = 0.001) at 3 months, 2.996° (95% CI 1.358–4.634; *p* < 0.001) at 6 months, and 2.353° (95% CI 0.699–4.008; *p* = 0.005) at 12 months. Similarly, the 30‐s chair‐stand test favored the guide‐plate group at all timepoints. The adjusted mean differences were 0.589 stands at 3 months (95% CI 0.200–0.977; *p* = 0.003), 0.836 stands at 6 months (95% CI 0.450–1.222; *p* < 0.001), and 0.705 stands at 12 months (95% CI 0.316–1.093; *p* < 0.001). At 3 months, the guide‐plate group also had significantly lower (better) scores on the WOMAC‐function subscale (adjusted mean difference −1.475, 95% CI –2.354 to 0.596; *p* = 0.001) and on the WOMAC‐stiffness subscale (adjusted mean difference −2.488, 95% CI –4.607 to 0.370; *p* = 0.021). No significant differences were observed at 6 or 12 months for WOMAC‐function or WOMAC‐stiffness (*p* > 0.05). The result of sensitivity analysis from per‐protocol population showed consistent results with intention‐to‐treat population (Tables S2 and S3).

**TABLE 3:** Changes in outcomes of guiding plate group and the control group at month 3, 6, and 12 (intention‐to‐treat analysis).

ITT^a^	3‐month			6‐month			12‐month		
	Guide plate (*N* = 90)	Control (*N* = 90)	*p*	Guide plate (*N* = 90)	Control (*N* = 90)	*p*	Guide plate (*N* = 90)	Control (*N* = 90)	*p*
Mechanical axis deviation (MAD) (mm)	−28.98 (4.12)	−27.80 (4.69)	0.086	−28.88 (4.03)	−27.88 (4.74)	0.142	−28.90 (4.12)	−27.99 (4.72)	0.187
ROM of knee flexion to extension (°)	9.82 (10.57)	3.61 (11.46)	0.000	14.35 (11.00)	10.95 (13.21)	0.072	16.83 (12.53)	16.02 (13.01)	0.686
Isometric knee flexion strength (% of unaffected side)	10.33 (2.19)	9.86 (1.70)	0.117	15.33 (2.19)	14.92 (1.85)	0.184	13.23 (2.25)	13.14 (2.61)	0.819
Dynamic knee flexion strength (% of unaffected side)	10.08 (1.88)	9.82 (2.22)	0.408	13.06 (2.06)	12.79 (2.23)	0.410	11.10 (2.04)	10.65 (2.51)	0.212
30‐s chair sit‐to‐stand test (times)	2.94 (0.50)	1.47 (1.77)	0.000	3.93 (0.74)	2.39 (1.70)	0.000	2.87 (1.31)	2.33 (1.79)	0.028
WOMAC‐pain	−24.58 (3.40)	−21.13 (4.35)	0.000	−39.29 (4.26)	−39.88 (5.34)	0.424	−41.52 (4.89)	−37.95 (15.30)	0.046
WOMAC‐function	−22.13 (3.14)	−18.54 (4.28)	0.000	−39.45 (3.16)	−39.40 (4.49)	0.930	−41.17 (3.47)	−36.89 (15.42)	0.015
WOMAC‐stiffness	−19.80 (6.20)	−14.40 (5.34)	0.000	−31.98 (6.24)	−31.64 (6.89)	0.737	−38.26 (9.08)	−34.31 (17.11)	0.067
Lysholm knee score	9.90 (1.08)	7.92 (1.13)	0.000	13.93 (1.34)	12.89 (1.19)	0.000	15.87 (1.29)	16.11 (1.25)	0.222
EQ‐5D‐5L‐utility	0.06 (0.08)	0.06 (0.06)	0.995	0.19 (0.10)	0.21 (0.10)	0.230	0.33 (0.11)	0.33 (0.11)	0.765

Abbreviations: EuroQol‐5 Dimension, 5‐Level; ITT, intention‐to‐treat; PROMs, patient‐reported outcome measures; ROM, range of motion; WOMAC, Western Ontario and McMaster Universities Osteoarthritis Index.

^a^ Values represent the mean change from baseline for each group, reported as mean (standard deviation), with *p* values comparing between‐group differences at each follow‐up point. All outcome measures were unadjusted. At 3‐, 6‐, and 12‐month follow‐ups, the number of participants with available data in the guide‐plate versus control groups were 88 versus 87, 87 versus 85, and 84 versus 85, respectively.

**TABLE 4:** Effectiveness estimates from linear mixed effects models of the guiding plate group and the control group at month 3, 6, and 12 (intention‐to‐treat analysis).

ITT^a^	3‐month			6‐month			12‐month		
	Coefficient	95% CI	*p*	Coefficient	95% CI	*p*	Coefficient	95% CI	*p*
Mechanical axis deviation (MAD) (mm)	−0.186	(−0.948, 0.576)	0.632	−0.399	(−0.975, 0.177)	0.175	−0.481	(−0.969, 0.006)	0.053
ROM of knee flexion to extension (°)	2.686	(1.120, 4.251)	0.001	2.996	(1.358, 4.634)	0.000	2.353	(0.699, 4.008)	0.005
Isometric knee flexion strength (% of unaffected side)	0.464	(−0.947, 1.875)	0.519	0.487	(−0.948, 1.921)	0.506	0.407	(−0.999, 1.812)	0.571
Dynamic knee flexion strength (% of unaffected side)	−0.649	(−2.131, 0.833)	0.391	−0.491	(−1.953, 0.971)	0.510	−0.436	(−1.875, 1.004)	0.553
30‐s chair sit‐to‐stand test (times)	0.589	(0.200, 0.977)	0.003	0.836	(0.450, 1.222)	0.000	0.705	(0.316, 1.093)	0.000
WOMAC‐pain	−1.541	(−3.751, 0.668)	0.171	−0.843	(−3.071, 1.384)	0.458	−0.591	(−2.758, 1.576)	0.593
WOMAC‐function	−1.475	(−2.354, −0.596)	0.001	−0.994	(−2.033, 0.046)	0.061	−0.792	(−1.815, 0.231)	0.129
WOMAC‐stiffness	−2.488	(−4.607, −0.370)	0.021	−1.817	(−4.027, 0.394)	0.107	−1.387	(−3.571, 0.798)	0.213
Lysholm knee score	1.330	(−0.223, 2.883)	0.093	1.254	(−0.293, 2.802)	0.112	0.890	(−0.619, 2.398)	0.248
EQ‐5D‐5L‐utility	0.001	(−0.022, 0.024)	0.937	−0.008	(−0.033, 0.017)	0.541	−0.008	(−0.033, 0.018)	0.545

Abbreviations: EuroQol‐5 Dimension, 5‐Level; ITT, intention‐to‐treat; PROMs, patient‐reported outcome measures; ROM, range of motion; WOMAC, Western Ontario and McMaster Universities Osteoarthritis Index.

^a^ Each coefficient represents the estimated between‐group difference in the change from baseline (Guide Plate group minus Control group) for the specified outcome at that follow‐up time point. Positive coefficients indicate higher scores in the Guide Plate group compared to the Control group, whereas negative values indicate lower scores in the Guide Plate group. Each estimate is presented with its 95% confidence interval (CI) and corresponding *p* value. All outcome measures were adjusted for baseline values in the model. At 3‐, 6‐, and 12‐month follow‐ups, the number of participants with available data in the guide‐plate versus control groups were 88 versus 87, 87 versus 85, and 84 versus 85, respectively.

### 3.3 Cost Measures

In the ITT analysis, direct medical costs for hospital stay, primary/secondary care and physical therapy did not differ significantly between groups. Implantation/device/medication cost was significantly higher in the guide‐plate arm (30,209 vs. 24,981 CNY, *p* = 0.000). Direct non‐medical costs (transportation, nutrition) and opportunity costs (lost wages) likewise showed no significant differences. Total cost did not differ significantly. The incremental total cost (guide plate vs. control) was 4373 CNY. Incremental outcomes were −0.481 mm for mechanical axis deviation (MAD) (ICER −9091 CNY per mm), +2.353° for knee flexion ROM (ICER 1858 CNY/°), +0.407% for isometric knee flexion strength (ICER 10,744 CNY/%), and −0.436% for dynamic flexion strength (ICER –10,030 CNY/%). The 30‐s chair stand test increased by +0.705 repetitions (ICER 6203 CNY/rep). For patient‐reported outcomes, incremental changes were −0.591 for WOMAC‐pain, −0.792 for WOMAC‐function, and −1.387 for WOMAC‐stiffness (ICERs −7399, −5521, and −3153 CNY/point, respectively). The Lysholm knee score increased +0.890 (ICER 4913 CNY/point) and EQ‐5D‐5L utility changed −0.008 (ICER –546,625 CNY). The per‐protocol (PP) analysis yielded similar ICERs; only total cost was significantly higher in the guide‐plate group (100,311 vs. 94,729 CNY, *p* = 0.037). The cost‐effectiveness acceptability curves for various indicators, comparing the Guide Plate and Conventional treatment strategies, was shown in Figures S1 and S2. Supplementary S3 and S4 present a probabilistic sensitivity analysis across different metrics, illustrating the distribution of ICERs for the Guide Plate and Conventional treatment strategies under varying incremental costs and benefits (Tables 5 and 6).

**TABLE 5:** Costs of guide plate group and the control group at month 12 (intention‐to‐treat analysis).

ITT	Guide plate (*N* = 90)	Control (*N* = 90)	*p*
Direct medical cost (CNY)			
Hospital stays cost (CNY)	7037 (1021.91)	7024 (1030.71)	0.935
Primary care cost (CNY)	2424 (527.88)	2477 (543.77)	0.528
Secondary care cost (CNY)	3775 (3297.67)	3816 (3194.90)	0.935
Physical therapist cost (CNY)	13,598 (1944.20)	13,254 (1973.69)	0.262
Implantation/device/medication (CNY)	30,209 (5014.28)	24,981 (4439.53)	0.000
Direct non‐medical cost (CNY)			
Transportation cost (CNY)	1964 (296.65)	1967 (294.64)	0.950
Nutrition cost (CNY)	4532 (459.52)	4536 (517.33)	0.962
Opportunity cost (CNY)			
Lost wages for patients (CNY)	33,363 (16,058.55)	34,652 (15,004.05)	0.595
Lost wages for families (CNY)	3362 (4378.05)	3184 (3743.96)	0.780
Total cost (CNY)	100,264 (17,331.33)	95,891 (16,007.01)	0.094

*Note*: All relevant costs were captured from a societal perspective. This included direct medical costs (Hospital stay, primary and secondary care, rehabilitation and physical therapy costs and medication costs), direct non‐medical costs related to care (transportation for medical visits and any specialized nutritional support during recovery), and indirect costs due to productivity loss (lost wages for patients during recovery or disability, and lost income for family members/caregivers, if applicable). Costs were obtained from hospital billing records and patient self‐reports where needed (wage losses). All costs are reported in 2024 Chinese Yuan, as 2024 was the end of the enrollment period; costs incurred in earlier years were adjusted to 2024 price levels using the consumer price index. Costs and outcomes occurring beyond the initial year were discounted at an annual rate of 3% to reflect time preference, consistent with standard practice in health economic evaluations. At 12‐month follow‐ups, the number of participants with available data in the guide plate versus control groups was 84 versus 85, respectively.

Abbreviations: CNY, Chinese Yuan; ITT, intention‐to‐treat.

**TABLE 6:** Incremental cost‐effectiveness ratio (ICER).

	ITT population	PP population
Incremental outcomes		
Incremental mechanical axis deviation (MAD) (mm)	−0.481 (−0.969, 0.006)	−0.679 (−1.123, −0.236)
Incremental ROM of knee flexion to extension (°)	2.353 (0.699, 4.008)	2.368 (0.645, 4.090)
Incremental isometric knee flexion strength (% of unaffected side)	0.407 (−0.999, 1.812)	0.315 (−1.179, 1.809)
Incremental dynamic knee flexion strength (% of unaffected side)	−0.436 (−1.875, 1.004)	−0.030 (−1.466, 1.407)
Incremental 30‐s chair sit‐to‐stand test (times)	0.705 (0.316, 1.093)	0.732 (0.318, 1.146)
Incremental WOMAC‐pain	−0.591 (−2.758, 1.576)	−0.591 (−2.758, 1.576)
Incremental WOMAC‐function	−0.792 (−1.815, 0.231)	−0.792 (−1.815, 0.231)
Incremental WOMAC‐stiffness	−1.387 (−3.571, 0.798)	−1.387 (−3.571, 0.798)
Incremental Lysholm knee score	0.890 (−0.619, 2.398)	0.675 (−0.892, 2.243)
Incremental EQ‐5D‐5L‐utility	−0.008 (−0.033, 0.018)	−0.006 (−0.033, 0.021)
ICER (CNY)		
Incremental cost	4373	5582
Mechanical axis deviation (MAD) (mm)	−9091	−8221
ROM of knee flexion to extension (°)	1858	2357
Isometric knee flexion strength (% of unaffected side)	10,744	17,721
Dynamic knee flexion strength (% of unaffected side)	−10,030	−186,067
30‐s chair sit‐to‐stand test (times)	6203	7626
WOMAC‐pain	−7399	−9445
WOMAC‐function	−5521	−7048
WOMAC‐stiffness	−3153	−4025
Lysholm knee score	4913	8270
EQ‐5D‐5L	−546,625	−930,333

*Note*: The table displays the incremental cost, incremental effect in primary outcome scores, and the resulting incremental cost‐effectiveness ratio (ICER). ICER is defined as the incremental cost divided by the incremental effect, representing the additional cost per unit improvement in outcome for the guide plate group versus the control group. Incremental effects are given for all outcome measures. ICER values are presented for the 2‐week follow‐up under both the intention‐to‐treat and per‐protocol analyses. A negative ICER indicates that the guide plate intervention achieved equivalent or better outcomes at a lower cost compared to control group.

Abbreviations: CHY, Chinese Yuan; EuroQol‐5 Dimension, 5‐Level; ICER, incremental cost‐effectiveness ratio; ITT, intention‐to‐treat; PP, per‐protocol; PROMs, patient‐reported outcome measures; ROM, range of motion; WOMAC, Western Ontario and McMaster Universities Osteoarthritis Index.

### 3.4 Adherence, Adverse Events, and Serious Adverse Events

Adherence to the rehabilitation program was similarly high in both groups regarding the numbers of exercise completed, self‐examination performed (Table 7). In the intention‐to‐treat population, 18 (20%) versus 20 (22%) patients experienced AEs in the guide‐plate versus control groups. Common AEs included surgery‐related events (lateral tibial fractures, superficial infections), rehab‐related issues (pain, swelling, muscle strain, arthromeningitis), and infrequent unrelated events (e.g., anxiety). SAEs occurred in 4 (4.4%) versus 3 (3.3%) patients; examples included one deep infection and one exercise‐related (waist) fracture in the guide‐plate group, and one nonunion and one hip fracture in the control group (Table S5).

**TABLE 7:** Adherence to rehabilitation program (intention‐to‐treat analysis).

ITT	Guide plate (*N* = 90)	Control (*N* = 90)	*p*
Completion of daily exercise session (times, maximum to 84)	66.3 (12.2)	65.9 (12.5)	0.653
Completion of weekly self‐examination (times, maximum to 12)	9.0 (2.2)	8.9 (2.3)	0.888
Number of receiving follow‐up phone calls (times, maximum to 4)	3.3 (0.7)	3.4 (0.8)	0.338
Number of follow‐ups in outpatient clinic (times, maximum to 4)	3.4 (0.7)	3.4 (0.6)	0.887

*Note*: Adherence data are reported as mean (standard deviations). Adherence data were retrieved from patients' logbooks and HIS (health information system) in both groups.

Abbreviation: ITT, intention‐to‐treat.

## 4 Discussion

### 4.1 General Findings of the Study

This multicenter randomized controlled trial evaluated the clinical and economic outcomes of 3D‐printed PSI guide plates in open‐wedge high tibial osteotomy. While the primary outcome of pain improvement did not differ significantly between groups, patients in the guide‐plate group showed modest advantages in certain functional outcomes, including range of motion and lower‐limb performance. Cost‐effectiveness analysis suggested that the guide plate approach incurred higher device‐related costs but yielded favorable incremental cost‐effectiveness ratios in some domains. Importantly, rehabilitation adherence was similarly high across both groups, and no major differences in adverse event rates were observed. These findings suggest that the PSI guide plate may offer technical and functional benefits without introducing additional safety concerns, though its overall clinical impact remains nuanced.

### 4.2 Interpretation of the Results

In line with our findings, several studies report that 3D‐printed PSI can achieve very close adherence to the preoperative plan. For example, Fucentese et al. [27] found mean deviations of only 0.8° in mechanical leg alignment and minimal unintended slope change when using PSI. Yang et al. [8] likewise reported that PSI‐guided HTO achieved planned weight‐bearing line correction with good tibial slope maintenance, and noted that the PSI workflow was precise, time‐saving, and reduced fluoroscopy use. These technical benefits must be balanced against clinical outcomes. For instance, Grillo et al. [28] observed that, despite shorter radiation exposure and similar operative times, the PSI group did not show superior alignment or patient‐reported scores (KOOS) at 3–12 months compared to conventional HTO. Similarly, Miao et al. confirmed in cadavers that 3D guides reproducibly execute the planned osteotomy without extra soft‐tissue stripping, but emphasized that careful technique is needed to avoid complications (e.g., lateral hinge fractures) [28]. On the other hand, MacLeod's computational study found that personalized HTO implants did not increase implant stress or failure risk versus generic designs, suggesting no new mechanical harms [29].

Taken together, the literature suggests that 3D‐printed PSI in HTO improves surgical precision and may streamline the procedure, but this has not yet translated into clear gains in long‐term function or pain relief [30]. Any potential advantages must therefore be weighed against the additional planning time, cost, and learning curve, as well as the small risk of guide‐related complications [31].

Our findings can be further contextualized by considering navigation‐assisted high tibial osteotomy, another technology‐driven approach aimed at improving accuracy. The evidence consistently indicates that computer navigation enhances the precision of alignment correction in OWHTO. Navigated procedures produce fewer outliers from the target mechanical axis and better maintain the posterior tibial slope compared to freehand techniques [32, 33]. Notably, the need for intraoperative fluoroscopy is markedly reduced under navigation guidance; one study reported the fluoroscopy time to be less than half of that in conventional OWHTO (10.4 vs. 24.8 s), which translates into substantially lower radiation exposure for the surgical team and patient [33]. However, these technical gains have not yielded superior clinical outcomes. A comprehensive meta‐analysis found that although navigation improved alignment accuracy, there were no significant differences in functional scores (Lysholm, IKDC, or KSS) or pain relief between navigated and conventional OWHTO [32, 33]. In fact, short‐term patient‐reported outcomes in navigated groups have mirrored those of standard surgery, echoing our trial's result that improved alignment alone may not improve pain at 1 year. Some analyses have noted a slight increase in knee range‐of‐motion among navigated OWHTO patients (on the order of 5°–6°), but this has not corresponded to meaningful differences in overall knee function or activity levels [32]. Navigation also entails additional setup: on average, operative time is about 10–15 min longer with navigation assistance, although at least one series reported that with an optimized technique the time difference was not significant [32, 33]. In summary, the experience with computer‐assisted OWHTO closely parallels our observations with PSI guides; both technologies improve surgical precision and reduce fluoroscopy requirements, yet neither has definitively proven an early clinical advantage over conventional methods.

### 4.3 Clinical Implication

Overall, our findings reinforce the growing consensus that while advanced guidance techniques like navigation or PSI improve the technical accuracy of OWHTO, they do not dramatically alter patient outcomes in the short term [34]. The potential value of this technology lies in its ability to translate preoperative plans into accurate osteotomy execution, which can enhance alignment accuracy and reproducibility, benefits that may be especially useful in complex cases requiring maximal precision. However, widespread adoption of patient‐specific guides faces significant challenges. The need for preoperative CT‐based planning, 3D printing, and surgeon training adds cost and time, and our trial (in line with previous studies) did not demonstrate any major short‐term advantage in pain relief or overall function compared to conventional techniques. In fact, aside from modest gains in knee flexion and performance tests, patient outcomes and cost‐effectiveness were similar between the PSI‐assisted and standard OWHTO groups at 12 months. Given the absence of clear early clinical superiority and the added resource burden, the use of 3D‐printed PSI in OWHTO is not yet justified on a broad scale. It may be best reserved for select scenarios, for instance, anatomically challenging deformities or cases where an exceptionally high degree of accuracy is deemed critical, until further evidence emerges. Looking ahead, future research will be crucial to clarify the role of this technology. Longer‐term follow‐up studies are needed to determine whether the improved alignment precision afforded by PSI leads to better durable outcomes, such as sustained cartilage preservation, lower rates of osteoarthritis progression, or reduced need for revision surgeries, over many years. Studies involving more diverse patient populations (including older individuals, higher BMI patients, or those requiring larger corrections with bone grafting) will help establish whether certain subgroups benefit more markedly from PSI‐guided osteotomy. Additionally, ongoing technical refinements aimed at streamlining the planning and fabrication process and reducing costs could improve the practicality and cost‐effectiveness of PSI [13, 35]. Until such advancements and evidence are in place, we advise a measured outlook: 3D‐printed patient‐specific guide plates should be applied judiciously, with optimism for their potential benefits tempered by recognition of their current limitations and the need for further validation through robust clinical research.

### 4.4 Strengths and Limitations

Major strengths include the rigorous multicenter randomized design and comprehensive societal‐perspective cost‐effectiveness analysis. Both groups followed a standardized postoperative rehabilitation protocol with high adherence. However, limitations include the confinement to two Chinese academic centers, potentially limiting generalizability to other settings or populations. The 12‐month follow‐up provides only intermediate‐term data, precluding assessment of longer‐term outcomes. The trial also did not collect detailed radiographic outcomes, safety metrics nor systematically track lateral hinge fracture rates, since our focus was on clinical and economic endpoints. The absence of these technical and safety outcomes is a limitation, as such measures are important indicators of correction accuracy and surgical precision. Future studies should incorporate these radiographic and technical parameters to fully evaluate the alignment benefits and safety of PSI‐guided HTO. Finally, the patient‐specific guide technique involves a learning curve and additional planning/training, which may constrain broader adoption. Finally, our patient population's characteristics limit the generalizability of these results. The trial cohort comprised relatively young adults (18–55 years old) with Kellgren–Lawrence grade II–III medial compartment OA and a predominantly lean BMI profile, reflecting an idealized HTO candidate group. This narrow selection does not capture the full spectrum of HTO patients seen globally. Many HTO patients worldwide are older or have higher body mass index values, and some present with concomitant lateral or patellofemoral compartment degeneration. Furthermore, no bone graft or substitute was used in any of our osteotomies, relying solely on stable plate fixation for gap healing. Although successful union and long‐term outcomes have been reported with graft‐free OWHTO in carefully selected cases, in routine practice surgeons often employ grafts or bone substitutes for larger corrections or poor bone stock. Therefore, the omission of grafting in our study and the youthful, active profile of our cohort narrow the applicability of these results. Our data should be extrapolated with caution to older, higher‐BMI, or multi‐compartment disease patients, as well as to cases requiring grafting for defect filling.

## 5 Conclusion

In this multicenter RCT of open‐wedge high tibial osteotomy, the 3D‐printed PSI guide plate did not provide superior pain relief compared to the conventional technique, and pain outcomes remained similar between groups. Some functional outcomes, notably knee range of motion and lower‐limb performance tests, were modestly improved with the guide plate. However, these gains came with higher device‐related costs and no clear overall cost‐effectiveness advantage. Overall, the findings indicate limited functional gain and no additional improvement in pain or cost‐effectiveness outcomes, suggesting any clinical benefit of the guide plate is likely modest.

## Author Contributions

**Runhua Zhou:** investigation, writing – original draft. **Yanjie Guo:** investigation, writing – original draft. **Ruixin Wang:** formal analysis. **Manrong Xu:** formal analysis. **Qinglin Kang:** investigation. **Yun Shen:** methodology. **Jia Xu:** investigation. **Da Zhong:** conceptualization, investigation, writing – review and editing. **Shengdi Lu:** conceptualization, methodology, writing – review and editing.

## Funding

The authors have nothing to report.

## Ethics Statement

The trial was approved by the Medical Ethics Committee of Xiangya Hospital Central South University (Approval number 202008253) and the Ethics Committee of Shanghai Sixth People's Hospital (Approval number 2020‐KY‐083(K)). All study procedures were conducted in accordance with the Declaration of Helsinki of 1964 and its later amendments.

## Consent

Written informed consent was obtained from all participants prior to enrollment, and participants were informed of their right to withdraw from the study at any time. All personal data were anonymized (de‐identified) to ensure participant privacy and confidentiality; therefore, specific consent to publish such data was not required. No financial or other compensation was provided to participants for their participation in this study.

## Conflicts of Interest

The authors declare no conflicts of interest.

## Supporting information


**Figure S1:** This figure presents the cost‐effectiveness acceptability curves for various indicators in the ITT analysis, comparing the Guide Plate and Conventional treatment strategies. It illustrates the probability (vertical axis) of each strategy being deemed cost‐effective at different willingness‐to‐pay (WTP) thresholds (horizontal axis). The horizontal axis ranges from 1 to 3 times China's per capita GDP in 2024. The trend of the curves reflects the dynamic changes in cost‐effectiveness advantages between the two strategies as the WTP threshold varies. When one curve lies above the other at a given WTP threshold, the corresponding strategy is more likely to demonstrate cost‐effectiveness advantages under that specific willingness‐to‐pay scenario.

**Figure S2:** This figure presents the cost‐effectiveness acceptability curves for various indicators in the PP analysis, comparing the Guide Plate and Conventional treatment strategies. It illustrates the probability (vertical axis) of each strategy being deemed cost‐effective at different willingness‐to‐pay (WTP) thresholds (horizontal axis). The horizontal axis ranges from 1 to 3 times China's per capita GDP in 2024. The trend of the curves reflects the dynamic changes in cost‐effectiveness advantages between the two strategies as the WTP threshold varies. When one curve lies above the other at a given WTP threshold, the corresponding strategy is more likely to demonstrate cost‐effectiveness advantages under that specific willingness‐to‐pay scenario.

**Figure S3:** This figure presents a probabilistic sensitivity analysis of the ITT set across different metrics, illustrating the distribution of ICERs for the Guide Plate and Conventional treatment strategies under varying incremental costs and benefits. Using the incremental benefits of outcomes from the baseline regression as a reference, sampling was performed with a normal distribution. Similarly, the total costs of the two treatment strategies from the baseline regression were used as a reference, with sampling conducted via a gamma distribution. The sampling process was repeated 5000 times to generate the scatter plot.

**Figure 4** This figure presents a probabilistic sensitivity analysis of various indicators for the PP set, illustrating the distribution of ICER for the Guide Plate and Conventional treatment strategies under different incremental costs and benefits. Using the incremental benefits of outcomes from the baseline regression as a reference, sampling was performed based on a normal distribution. Similarly, the total costs of the two treatment strategies from the baseline regression were used as a reference, with sampling conducted via a gamma distribution. The sampling process was repeated 5000 times, and the results were plotted as a scatter diagram.

**Figure S5:** This figure shows the design of the 3D‐Printed Patient‐Specific Guide Plate, including the drill guide holes for Kirschner wires and a cutting slot (left, with an anterior guiding plane) to control the trajectory and depth of the oscillating saw blade during the biplanar osteotomy, as well as a modular wedge spacer component (right) that set the opening gap distance corresponding to the planned correction.

**Data S1:** os70259‐sup‐0006‐SupplementaryMaterials.docx.

## Data Availability

The data that support the findings of this study are available on request from the corresponding author. The data are not publicly available due to privacy or ethical restrictions.
