# Supplementary figures and images for: The Efficiency and Cost‐Effectiveness of 3D‐Printed Patient‐Specific Guide Plate for Patients Undergoing Open‐Wedge High Tibial Osteotomy: A Multicentered Randomized Controlled Trial
Source: Orthop Surg. 2026 Feb 15;18(3):474–88. doi: 10.1111/os.70259 (PMC12967555; doi:10.1111/os.70259)

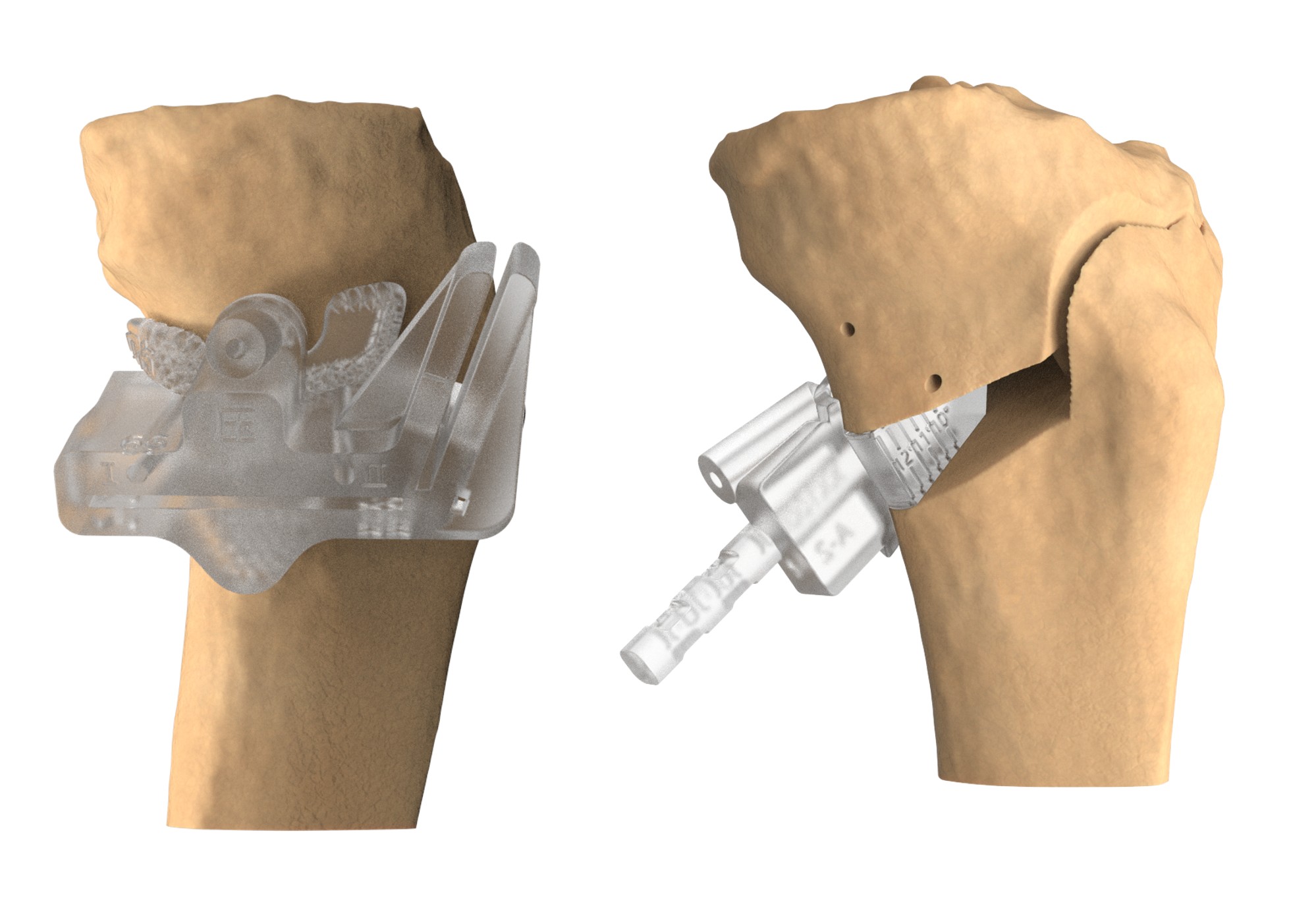

Supplement: Supplementary file 5 — Figure S5: This figure shows the design of the 3D‐Printed Patient‐Specific Guide Plate, including the drill guide holes for Kirschner wires and a cutting slot (left, with an anterior guiding plane) to control the trajectory and depth of the oscillating saw blade during the biplanar osteotomy, as well as a modular wedge spacer component (right) that set the opening gap distance corresponding to the planned correction. [file OS-18-474-s002.jpg]
