# Supplementary material for: The Efficiency and Cost‐Effectiveness of 3D‐Printed Patient‐Specific Guide Plate for Patients Undergoing Open‐Wedge High Tibial Osteotomy: A Multicentered Randomized Controlled Trial
Source: Orthop Surg. 2026 Feb 15;18(3):474–88. doi: 10.1111/os.70259 (PMC12967555; doi:10.1111/os.70259)
Supplement: Supplementary file 6 — Data S1: os70259‐sup‐0006‐SupplementaryMaterials.docx. [file OS-18-474-s006.docx]

**Supplementary Materials**

**Intervention**

**Design and Validation of 3D-Printed Patient-Specific Guide Plate**

In the guide plate group, each patient received a custom-designed osteotomy guide plate based on their own preoperative CT anatomy. Patient CT DICOM data were segmented with Mimics software to create a 3D model of the proximal tibia. A computer simulation incorporating finite element modeling was used during planning to optimize the osteotomy configuration, determining the ideal osteotomy level, hinge position, and correction angle required to achieve the target realignment while minimizing stress at the lateral hinge. The guide plate was then virtually designed to fit the patient’s bony surface and included precise built-in slots for guiding instrumentation. Specifically, it featured dedicated drill guide holes for Kirschner wires and a cutting slot (with an anterior guiding plane) to control the trajectory and depth of the oscillating saw blade during the biplanar osteotomy. The design also incorporated a modular wedge spacer component that set the opening gap distance corresponding to the planned correction; this spacer could be inserted into the osteotomy site to maintain the desired distraction height and angular correction until internal fixation was applied. Throughout the design process, we established the key parameters: the osteotomy plane orientation, the location of guide placement, and the size of any wedge or gap necessary to achieve the mechanical axis realignment. We also continually performed virtual fit checks: the guide model was virtually “test-fitted” onto the 3D bone model to confirm that it conformed perfectly to the bone and that all guide features (slots and holes) aligned with the intended anatomical landmarks and osteotomy plane. This digital validation step was crucial, it ensured that the custom guide would sit flush and stable on the patient’s bone and cut in the correct orientation before we moved on to fabrication. By virtually simulating the guide placement and even a mock osteotomy on the computer model, we could verify the accuracy of our design and make any fine adjustments in silico, rather than discovering issues in the operating room. This rigorous design and validation workflow gave us confidence that the final guide would translate the planned correction to the patient’s anatomy with high fidelity.

Once the design was finalized and verified, the guide was manufactured using 3D printing technology. We utilized a high-resolution stereolithography (SLA) 3D printer to fabricate the guide. In our case, the guide was printed on a Form 2 SLA printer (Formlabs Inc., Somerville, MA, USA) using a biocompatible photopolymer resin. This printer offers an in-plane resolution of about 25 µm, which allowed us to capture fine details of the guide, such as precise cutting slot geometry and snug-fitting peg holes. The print file (STL of the guide) was processed with the printer’s software (PreForm) to generate support structures and sliced into layers. We selected a layer thickness of 0.05–0.1 mm during printing, a setting that provides an excellent balance between detail and fabrication time for surgical guides. The SLA printing process then built the guide layer-by-layer from the liquid resin, faithfully reproducing the complex geometry of the patient-specific design. After printing, the guide underwent standard post-processing: it was gently cleaned of residual resin and cured under UV light to reach its full strength and material stability. The resulting polymer guide was rigid and accurate; importantly, the resin we used is rated as a Class I biocompatible material, which means the printed guide can safely contact human tissue and withstand common sterilization methods. Prior to surgery, the guide was sterilized (using low-temperature gas plasma sterilization in our protocol) without any significant warping or dimensional change, as the material and printing parameters were chosen to minimize thermal distortion The more detailed design of and surgical application of the 3D-Printed Patient-Specific Guide Plate was presented in relevant literatures (Gao F, et al. Comparison of Clinical and Radiological Outcomes between Calibratable Patient-Specific Instrumentation and Conventional Operation for Medial Open-Wedge High Tibial Osteotomy: A Randomized Controlled Trial. Biomed Res Int. 2022; 2022:1378042.). Supplementary Figure 5 shows the design of the 3D-Printed Patient-Specific Guide Plate as well as its modular wedge spacer component.

**Surgical Procedure**

All procedures were performed under general anesthesia with the patient supine on a standard radiolucent operating table. A well-padded high-thigh tourniquet was applied to the operative leg, and the non-operative leg was secured in extension. The ipsilateral knee was positioned with a padded bolster to allow approximately 30° of flexion, facilitating access and fluoroscopic imaging. The limb was prepped and draped from mid-thigh to foot in the usual sterile fashion.

**Guide Plate group**

1. Preoperative planning and guide preparation: A thin-slice CT scan (1.0 mm) of the entire lower extremity was obtained and used to reconstruct a 3D tibial model in planning software (Mimics). The desired correction (mechanical axis target) and osteotomy planes were determined. A cutting guide and corresponding opening wedge spacer were designed on the medial proximal tibia to achieve this correction. These patient-specific templates (with drill holes for K-wires and saw slots for the osteotomy) were 3D-printed from biocompatible material and sterilized before surgery.
2. Exposure: With the tourniquet inflated, a medial anteromedial incision (6 to 8 cm long) was made midway between the tibial tubercle and the posteromedial tibial border. The pes anserinus tendons were identified and incised or reflected, and subperiosteal dissection exposed the medial tibial cortex. A retractor was placed to protect the patellar tendon anteriorly, and a Cobb or periosteal elevator was used on the posteromedial tibia to elevate the superficial medial collateral ligament (sMCL) and pes anserinus off the bone (with a radiolucent retractor protecting neurovascular structures). This ensured full exposure of the proximal medial tibia and lateral hinge area.
3. PSI guide placement and fixation: The sterilized cutting guide was seated on the medial tibia according to the preoperative plan. It was keyed to anteromedial contours and fixed in place with Kirschner wires through its proximal and distal pin holes. Fluoroscopy (AP and lateral) was used to confirm correct guide positioning and orientation in both coronal and sagittal planes.
4. Osteotomy through the guide: A biplanar osteotomy was performed along the guided slots. First, an oscillating saw was used to make a medial-to-lateral (horizontal) cut, aiming toward the lateral hinge but stopping approximately 1–2 mm short of the lateral cortex. Then an ascending (slanted) cut was made from the end of the first cut toward the anterior tibia (just beneath the patellar tendon insertion) to complete the biplanar osteotomy. Depth-limited osteotomes or chisels were used to carry these cuts toward the lateral hinge, preserving a thin cortex. Next, laminar spreaders were inserted sequentially into the posterior and anterior gaps to gradually open the osteotomy to the planned correction. The opening was controlled and held (often with an inserted wedge or calibrated spacer block) matching the preplanned wedge height. A custom angular spacer (depth gauge) was placed in the gap to achieve the target correction angle.
5. Fixation and closure: A contoured medial tibial locking plate (TomoFix) was applied over the opened osteotomy and provisionally held in place (often with a K-wire). Locking screws were inserted in standard sequence (proximal fragment first, then distal fragment) under fluoroscopic guidance, securing the osteotomy in the corrected alignment. Final anteroposterior and lateral radiographs confirmed satisfactory alignment and hinge integrity. The wound was closed in layers in the usual fashion, and the tourniquet was released.

**Conventional freehand (Control) group**

1. Exposure: The surgical approach was the same as above. A medial anteromedial incision (approximately 6–8 cm) was made between the tibial tubercle and posteromedial border. The pes anserinus and superficial MCL were dissected and elevated subperiosteally in the same manner, with retractors protecting the patellar tendon and MCL as needed.
2. Guide-wire placement and osteotomy: Under biplanar fluoroscopy, one or two guide pins (K-wires) were placed from the medial tibial cortex toward the lateral hinge (proximal pins aiming just above the fibular head) to outline the osteotomy plane. These wires served as visual references for the cuts. A horizontal saw cut was made freehand along the planned plane between the wires, again preserving the lateral cortex. An ascending cut was then performed from the end of the horizontal cut toward the anterior tibia, creating a biplanar osteotomy. The guide wires in situ helped maintain proper trajectory of the cuts.
3. Opening and fixation: Laminar spreaders were inserted as above to open the medial wedge gradually until the desired valgus correction was reached. Bone graft or synthetic wedge material could be placed in the gap to maintain the opening if desired. A medial locking plate was then applied and fixed with locking screws in the standard manner (proximal screws first, then distal), identical to the PSI group fixation. Fluoroscopy confirmed correction and hardware position before final wound closure.

**Postoperative Care and Rehabilitation Protocol**

**Postoperative Care**

A multimodal approach is provided, including scheduled non-opioid analgesics, appropriate prophylaxis, and physical measures. The key components are outlined below:

Pain Management

1. Regular non-opioid analgesics: Give scheduled acetaminophen (paracetamol) and a nonsteroidal anti-inflammatory (ibuprofen). Combining acetaminophen and an NSAID significantly improves pain control and reduces the need for opioids.
2. Opioids for breakthrough pain: Reserve short-acting opioids (oxycodone 5–10 mg orally) only for severe pain not relieved by non-opioids. Use the lowest effective dose for the shortest duration to limit side effects.
3. Neuropathic agents: If nerve-related pain is anticipated, a gabapentinoid (gabapentin or pregabalin) may be added perioperatively, as these can reduce opioid requirements.
4. Cryotherapy: Apply ice packs over the surgical site. Clinical studies show that cryotherapy substantially reduces postoperative pain and opioid use during the first 1–2 days.

Antibiotic Prophylaxis

Use the standard perioperative antibiotic (IV cefazolin before incision) according to protocol. Do not continue antibiotics beyond 24 hours after surgery. In fact, guidelines note that “most procedures require no post-op doses of antimicrobials,” and any additional prophylaxis should be limited to *<24 hours* post-closure.

Thromboprophylaxis

Pharmacologic VTE prophylaxis is indicated. For example, enoxaparin (30 mg SC twice daily) or rivaroxaban (10 mg PO daily) is given for at least 10–14 days, often extending to 30–35 days post-op. (Rivaroxaban is FDA-approved for DVT/PE prevention after hip or knee replacement.)

Elevation and Mobilization

1. Elevate the limb: Keep the operated leg/foot elevated above heart level when resting. Elevation (on pillows) helps drain fluid and reduce swelling, especially during the first 4–6 weeks post-op[.](https://pmc.ncbi.nlm.nih.gov/articles/PMC10573441/#:~:text=advised%20to%20rest%20and%20keep,NSAIDs%20after%20a%20few%20days).
2. Protect weight-bearing: Use crutches or a postoperative boot/cast as directed to maintain non-weightbearing initially. Begin gentle physical therapy as soon as appropriate (light range-of-motion and isometric exercises, lymphatic drainage techniques) to promote recovery and control swelling[.](https://pmc.ncbi.nlm.nih.gov/articles/PMC10573441/#:~:text=advised%20to%20rest%20and%20keep,NSAIDs%20after%20a%20few%20days).

**Rehabilitation Protocol**

| **Week** | **Assessment** | **Goals** | **Manual Therapy** | **Supervised Exercises (per session, frequency)** | **Modalities** | **Remarks** |
| --- | --- | --- | --- | --- | --- | --- |
| 1 | Check surgical wound, swelling, pain level; measure knee ROM and quadriceps activation. Gait/balance with device. | Protect incision; control pain and edema; achieve full extension (0°); increase flexion (30°). Activate quadriceps (SLR). | Patellar mobilizations (grade I–II); tibiofemoral glides for comfort; gentle soft-tissue massage (hamstring/quadriceps). | 4×/week: Ankle pumps, calf stretches; quadriceps sets; straight leg raise (with brace if lag); short-arc quad exercises; assisted heel slides to tolerance; bedside sitting and standing with support. | Cryotherapy (ice 15–20 min after sessions); compression and elevation as ordered. NMES on quadriceps during sets (if available). | Use walker/crutches for gait (partial or as tolerated). Avoid twisting. Ensure prone or supine positioning for exercises to maximize extension. Home exercises between sessions. |
| 2 | Reassess ROM, strength, pain. Check ability to do SLR without lag. Monitor gait with reduced assistance. | Increase flexion (60°); maintain full extension; improve quad control; begin weight-bearing tolerance. | Continue patellar and joint glides (progress grade as tolerated); soft-tissue mobilization of leg muscles. | 4×/week: Seated active-assisted ROM (heel slides, wall slides to 60°); bridging; supine knee extensions (heel propped) aiming for full extension; standing weight shifts; assisted sit-to-stand; patellar mobilization exercises. | Continue cryotherapy post-session. Continue NMES on quads to augment contraction. | Encourage walking with assistive device for short distances; adjust device if safe (downgrade from walker to front-wheeled walker). Monitor incision. |
| 3 | Assess ROM, pain, swelling, muscle tone. Test single-leg stance balance (assisted). | Achieve flexion 80°; full extension; improve static balance; begin dynamic tasks. | Manual glides to improve ROM if plateauing; gentle mobilization in flexion. Scar mobilization if needed (superficial). | 4×/week: Active-assisted heel slides (aim 80°); stationary bike (low resistance) for ROM; seated knee flexion/extension; partial squats (seated to standing), mini lunges (supportive); quadriceps and glute sets; single-leg stance (assisted). Gait training on level floor. | Cryotherapy as needed for pain. NMES as needed for quad. Begin electrotherapy for swelling control if prescribed. | Continue using device; try decreasing steps if stable. Educate patient on home ROM and gentle strengthening exercises. |
| 4 | Re-evaluate ROM, quadriceps MMT, gait pattern without limp. | Achieve flexion ≥90°; full extension; independent SLR; safe ambulation with device. | Continue mobilizations (tibiofemoral and patella) to sustain ROM gains; address any adhesions. | 4×/week: Advance heel slides/wall slides toward 90°; seated knee extensions (straightening) for end-range; stationary cycling (increase duration); sit-to-stand to full extension; step-ups (6" step); continued quad sets. Begin gentle hamstring curls. | Cryotherapy after exercise; begin heat (if swelling minimal) before sessions to aid stretching. | Wean brace (if used); progress from walker toward 2 canes as tolerated. Begin weaning assistive device when patient can ascend stairs step-over-step with minimal limp. |
| 5 | Test ROM, strength (MMT quads, hip), gait symmetry; assess balance (single-leg). | Achieve flexion 100°; near-normal extension; improve strength; independent ADLs. | Grade III patellar and tibiofemoral mobilizations to maximize ROM; soft-tissue release of quadriceps/iliotibial band if stiff. | 3×/week: Knee flexion/extension with light resistance (ankle weight); closed-chain exercises: mini-squats (to 30–45° flexion), leg press (light), heel raises; stationary bike (increase resistance); gait training including slight incline and decline; begin proprioceptive exercises (balance pad). | Pulsed ultrasound or heat before therapy if tolerated to loosen tissue; cryotherapy after. Continue NMES until strong volitional quad contraction achieved. | Consider weaning to single cane. Encourage walking on even surfaces without device if safe. Monitor for any gait deviations and correct. |
| 6 | Assess functional tasks: chair rise, stairs; strength (MMT 4/5 target for quads). | Achieve flexion ≥110°; independent stairs with railing; improve cardiovascular endurance. | Joint mobilizations as needed for lingering ROM deficits; address hip/pelvic alignment manually if needed. | 3×/week: Progress squats and lunges (supported weight increasing); lateral step-ups; sit-to-stand from lower chair; stationary bike (higher resistance); begin elliptical or treadmill walking; balance: tandem stance/walk. Hamstring curls with resistance band. | Continue modalities as needed: thermotherapy or ultrasound pre-session, cryotherapy post-session. | Transition to cane-free gait if able. Introduce pool therapy/aquatic walking if incision healed and available. Emphasize posture and core engagement. |
| 7 | Reassess any functional limitations. Test quad strength at 50% body weight. Evaluate walking endurance (6 min walk). | Full ROM (flexion 120°); ≥80% strength of opposite side; normal gait; single-leg balance 15 s. | Tackle any residual restrictions: deep flexion patellar mobilization; soft tissue work on calf and adductors. | 3×/week: Advance resistance: weight machines (leg extension/curl, leg press); multi-direction lunges; single-leg squats (supported); stair climbing; progressive ambulation (longer distances, variable speeds). | Continue strengthening modalities (NMES if needed on lagging side). Use of light weights or resistance tubing. | Incorporate light functional tasks: carrying objects while walking, turning. Ensure patient performs exercises correctly at home for maintenance. |
| 8 | Measure ROM, strength, functional performance (sit-to-stand test); gait on uneven ground. | Maintain full ROM; strengthen to daily functional level; improve balance/proprioception. | Maintenance mobilizations; trigger point release for calf/hip flexors if tight. | 3×/week: Begin dynamic balance (BOSU, wobble board); lateral movements (side steps); sport/activity-specific movements if indicated (mini hops); treadmill with incline. Continue leg strengthening and biking. | If available, proprioceptive or biofeedback training. | Prepare for discharge to 2×/week sessions. Encourage active home exercise program focusing on any deficits. |
| 9 | Evaluate endurance (6MWT), stair climb, balance. Compare strength to pre-op (target ≥80%). | Increase endurance and functional strength; safe community-level mobility. | As needed, mobilize joints at end-range to sustain flexibility. | 2×/week: Increase intensity: squats/lunges with weight; step-ups at 8" height; single-leg stance with perturbations; forward/backward walking; stationary bike (longer duration); add pool running/walking if possible. | Continue any supportive modalities (taping for patellar tracking if needed during activity). | Reinforce healthy gait mechanics. Address any compensations. |
| 10 | Check all criteria (ROM, strength, function); focus on any lagging deficits. | Achieve ≥90% strength of contralateral leg; full daily activities without pain or limp. | Manual therapy as adjunct if minor ROM issues remain. | 2×/week: Functional exercises: carrying moderate loads, uneven terrain walking; advanced balance tasks (single-leg eyes closed); continue gym equipment training. Encourage resistance training (leg press, squats) progressing to near full weight. | NMES can be discontinued if strength nearly normal. Use heat pre-session to optimize flexibility. | Continue to reduce therapy frequency if goals met, maintaining 2×/week. Encourage independent exercise adherence. |
| 11 | Final evaluation of deficits; patient readiness for independent exercise. | Finalize strength and proprioception for full return to activity. | Final joint mobilizations if needed for maximal ROM. | 2×/week: Community tasks: climbing stairs carrying load; step-downs from a step; continued strengthening with heavier resistance; cardiovascular exercise (treadmill, elliptical longer sessions). | No specific modalities unless patient has residual pain (use ice). | Ensure patient can perform all exercises independently. Plan long-term home exercise maintenance program. |
| 12 | Outcome measures: ROM, strength (target ≥90% nonoperative), 6MWT, patient-reported function. Final gait and balance check. | Maintain or exceed discharge goals; prepare for discharge from supervised therapy. | Only if minor issues, otherwise complete. | 2×/week: Repeat prior advanced exercises; transition sessions focus to patient’s activity goals (sports drills); introduce low-impact plyometrics if appropriate (heel raises into small hops). | Encourage ongoing self-therapy (home or community gym). | Discharge with a home exercise plan. Schedule follow-ups (6-month check) to ensure long-term adherence. |

**Regulation of the Rehabilitation Protocol:** Exercise prescriptions are individualized by the therapist based on each patient’s baseline status and weekly progress. For patients with poor preoperative mobility or weak musculature, initial exercises are more passive and assisted (for example, greater reliance on therapist support and NMES) with slower progression. Higher-functioning patients advance sooner to active strengthening and dynamic tasks. At each visit, therapists compare measured range of motion and strength to milestone targets (for example achieving knee flexion of 90° by week 4 or a straight-leg raise without lag); meeting these criteria triggers an increase in exercise resistance, range, or functional complexity. If pain, swelling, or deficits persist, progression is delayed and exercises are maintained at the current level or regressed (for example repeating earlier exercises or increasing manual therapy). Assistive device use is tapered when patients demonstrate stable gait and adequate quadriceps control; if balance or strength are insufficient, device use is continued longer. Overall, the protocol is dynamically adjusted to ensure safety while promoting maximal recovery of function.

**Statistical Analysis**

Baseline patient characteristics, outcomes, and treatment adherence were summarized using standard descriptive statistics. Categorical variables are reported as counts and percentages, and continuous variables are reported as means with standard deviations (SD). For each outcome at each time point, group means (SD) are presented, and between-group differences are expressed as estimated coefficients with two-sided 95% confidence intervals (CI). Statistical tests were two-sided and a significance level of 0.05 was used throughout.

The primary outcome (WOMAC pain score at 12 months post-randomization) and secondary outcomes were analyzed in the intention-to-treat (ITT) population, which includes all randomized participants according to their assigned treatment groups regardless of adherence or dropout. We used mixed-effects linear regression models to compare outcomes between the guide plate group and the control group. In these models, the outcome score at each time point was the dependent variable. Fixed effects included treatment group, follow-up time (as a categorical variable), and the interaction between treatment and time. The models were adjusted for baseline covariates: age, gender, and the baseline value of the outcome score. Random effects were specified for study center (to account for potential center-level differences) and for participant (to account for the repeated measurements within individuals). The treatment-by-time interaction term allows the estimated treatment effect to vary over the follow-up visits. From the mixed-effects model, we obtained adjusted estimates of the group differences at each time point along with their 95% CIs and P values. All reported P values for these analyses are two-sided.

The analysis of the primary and secondary outcomes was also repeated in the per-protocol (PP) population. The PP population included only those participants who completed all scheduled follow-up visits without major protocol deviations. This provides a sensitivity analysis to compare with the ITT results. In the PP analysis, because only complete cases (no missing visits) were included, the mixed-effects models were fitted in the same way but on this restricted dataset.

No imputation was performed for missing outcome data. Missing data occurred only at the visit level (participant dropout) and not at the item level within a completed visit. In the ITT approach, all available data for each participant were included in the mixed model, which uses maximum likelihood estimation under a “missing at random” assumption to handle intermittent missing outcomes without requiring imputation. In practical terms, if a participant missed a scheduled visit, all scores from that visit were absent, but their other time points were still used in the analysis. Because entire follow-up visits were missing for dropouts, standard imputation methods were not applied. We also note that a test of missing‐completely‐at‐random (such as Little’s MCAR test) was not applicable given the pattern of missingness (complete visits missing for dropouts, with no partial missingness within visits). We applied a false discovery rate correction using the Benjamini–Hochberg method to control for multiple comparisons across all secondary outcomes measured at 3, 6, and 12 months.

Cost-effectiveness was evaluated by computing the incremental cost-effectiveness ratio (ICER) of the guide plate intervention versus control. The ICER is defined as the ratio of the difference in mean cost to the difference in mean effectiveness between the two groups. To estimate uncertainty in the ICER, we used a non-parametric bootstrap approach with 1,000 replications. We performed the bootstrap on the multiple-imputed dataset. In each iteration, we randomly sampled with replacement the same number of patients as in the original trial from each group. For each bootstrap sample we calculated (1) the incremental cost (difference in mean total cost between guide plate and control, using costs observed at 12 months as the total cost for the period) and (2) the incremental effectiveness. The incremental effectiveness was computed for each outcome and each follow-up time point, specifically three outcome measures at three time points (yielding nine treatment differences), as the difference in mean outcome between groups. From the distribution of bootstrap estimates we derived confidence intervals for the incremental cost and the incremental effectiveness measures, and these were used to characterize the uncertainty in the ICER.

**References**

1. Gao F, Yang X, Wang C, Su S, Qi J, Li Z, Chen J, Zhong D. Comparison of Clinical and Radiological Outcomes between Calibratable Patient-Specific Instrumentation and Conventional Operation for Medial Open-Wedge High Tibial Osteotomy: A Randomized Controlled Trial. Biomed Res Int. 2022; 2022:1378042.
2. Adie S, Naylor JM, Harris IA. Cryotherapy after total knee arthroplasty: a systematic review and meta-analysis of randomized controlled trials. J Arthroplasty. 2010 Aug;25(5):709–15.
3. Bade MJ, Struessel T, Dayton MR, et al. Early high-intensity versus low-intensity rehabilitation after total knee arthroplasty: a randomized controlled trial. Arthritis Care Res (Hoboken). 2017 Sep;69(9):1360–8.
4. Petterson SC, Mizner RL, Stevens JE, et al. Improved function from progressive strengthening interventions after total knee arthroplasty: a randomized clinical trial with an embedded prospective cohort. Arthritis Rheum. 2009 Feb 15;61(2):174–83.
5. Peng L, Wang K, Zeng Y, et al. Effect of neuromuscular electrical stimulation after total knee arthroplasty: a systematic review and meta-analysis of randomized controlled trials. Front Med (Lausanne). 2021;8:779019.
6. Dávila Castrodad IM, Recai TM, Abraham MM, et al. Rehabilitation protocols following total knee arthroplasty: a review of study designs and outcome measures. Ann Transl Med. 2019 Oct;7(Suppl 7):S255.
7. Schulz KF, Altman DG, Moher D; CONSORT Group. CONSORT 2010 statement: updated guidelines for reporting parallel group randomised trials. BMC Med. 2010;8:18.
8. Gupta SK. Intention-to-treat concept: a review. Perspect Clin Res. 2011;2(3):109-12.
9. Holmberg MJ, Andersen LW. Adjustment for baseline characteristics in randomized clinical trials. JAMA. 2022;328(21):2155-56.
10. Austin PC, White IR, Lee DS, van Buuren S. Missing data in clinical research: a tutorial on multiple imputation. Can J Cardiol. 2021;37(9):1322-31.
11. Severens JL, de Boo TM, Konst EM. Uncertainty of incremental cost-effectiveness ratios: a comparison of Fieller and bootstrap confidence intervals. Int J Technol Assess Health Care. 1999;15(3):608-14.
12. Gibbons RD, Hedeker D, du Toit S. Advances in analysis of longitudinal data. Annu Rev Clin Psychol. 2010;6:79-107.
13. Little RJ, D’Agostino R, Cohen ML, et al. The prevention and treatment of missing data in clinical trials. N Engl J Med. 2012;367(14):1355-60.

Supplementary Table 1 Baseline characteristics of the guide plate group and the control group (per-protocol analysis)

| **PP** | **Characteristics*** | **Guide Plate (N=79)** | **Control (N=77)** | **P value** |
| --- | --- | --- | --- | --- |
| **Baseline Characteristics** | Male Gender (no. [%]) | 46 (58.23) | 45 (58.44) | 0.978 |
|  | Age (yrs) | 55 (2.87) | 55 (2.89) | 0.548 |
|  | BMI (kg/m²) | 23 (2.79) | 23 (2.88) | 0.916 |
|  | Occupation (no. [%]) |  |  | 0.750 |
|  | Manual worker | 38 (48.10) | 39 (50.65) |  |
|  | Non-manual worker | 41 (51.90) | 38 (49.35) |  |
|  | Education level (no. [%]) |  |  | 0.954 |
|  | Lower than high school | 70 (88.61) | 68 (88.31) |  |
|  | Equal/higher to high school | 9 (11.39) | 9 (11.69) |  |
|  | Insurance type (no. [%]) |  |  | 0.957 |
|  | Government | 71 (89.87) | 69 (89.61) |  |
|  | Commercial | 8 (10.13) | 8 (10.39) |  |
|  | Self-financed | 0 (0.00) | 0 (0.00) |  |
|  | Current Smoker (no. [%]) | 24 (30.38) | 20 (25.97) | 0.541 |
|  | Current Alcohol use (no. [%]) | 16 (20.25) | 15 (19.48) | 0.904 |
|  | Paracetamol and NSAIDs (no. [%]) | 20 (25.32) | 19 (24.68) | 0.926 |
|  | Living alone (no. [%]) | 26 (32.91) | 25 (32.47) | 0.953 |
|  | Walking aid (no. [%]) | 50 (63.29) | 52 (67.53) | 0.578 |
| **Comorbid illness** | Osteoporosis (no. [%]) | 52 (65.82) | 50 (64.94) | 0.907 |
|  | Hypertension (no. [%]) | 6 (7.59) | 5 (6.49) | 0.788 |
|  | Diabetes (no. [%]) | 10 (12.66) | 11 (14.29) | 0.766 |
|  | COPD (no. [%]) | 5 (6.33) | 5 (6.49) | 0.967 |
|  | Peripheral vascular disorder (no. [%]) | 5 (6.33) | 4 (5.19) | 0.761 |
|  | Arthritis in other joints (no. [%]) | 13 (16.46) | 11 (14.29) | 0.707 |
| **KOA severity** | Kellgren-Lawrence grade (no. [%]) |  |  | 0.770 |
|  | Grade II | 60 (75.95) | 60 (77.92) |  |
|  | Grade III | 19 (24.05) | 17 (22.08) |  |
|  | Patellofemoral OA (no. [%]) |  |  | 0.804 |
|  | Grade I | 55 (69.62) | 55 (71.43) |  |
|  | Grade II | 24 (30.38) | 22 (28.57) |  |
|  | Mechanical axis deviation (MAD) (mm) | 30 (4.06) | 30 (4.29) | 0.581 |
| **Knee Function** | ROM of knee flexion to extension (°) | 105 (7.88) | 105 (7.95) | 0.921 |
|  | Isometric Knee Flexion Strength (% of unaffected side) | 82 (4.98) | 82 (5.05) | 0.986 |
|  | Dynamic Knee Flexion Strength (% of unaffected side) | 85 (5.32) | 85 (4.35) | 0.776 |
|  | 30-s chair sit-to-stand test (times) | 11 (1.54) | 11 (1.49) | 0.456 |
| **PROMs** | WOMAC-pain | 61 (7.68) | 61 (7.26) | 0.937 |
|  | WOMAC-function | 59 (3.11) | 59 (3.34) | 0.796 |
|  | WOMAC-Stiffness | 56 (7.63) | 57 (7.91) | 0.885 |
|  | Lysholm Knee Score | 60 (5.15) | 60 (5.78) | 0.937 |
|  | EQ-5D-5L-utility | 0.35 (0.10) | 0.35 (0.09) | 0.845 |

PP: per-protocol; KOA: knee osteoarthritis; BMI: body mass index; NSAIDs: Non-Steroidal Anti-Inflammatory Drugs; COPD: Chronic Obstructive Pulmonary Disease; ROM: range of motion; PROMs: patient-reported outcome measures; WOMAC: Western Ontario and McMaster Universities Osteoarthritis Index; EuroQol-5 Dimension, 5-Level.

* Values were reported as mean (standard deviation) for age, BMI, Mechanical axis deviation (MAD), all variables of knee function and PROMs, others were reported as number (percentage).

Supplementary Table 2 Changes in outcomes of guiding plate group and the control group at month 3, 6 and 12 (per-protocol analysis)

| **PP^*^** | **3-month** | | | **6-month** | | | **12-month** | | |
| --- | --- | --- | --- | --- | --- | --- | --- | --- | --- |
|  | **Guide Plate (N=79)** | **Control (N=77)** | **P value** | **Guide Plate (N=79)** | **Control (N=77)** | **P value** | **Guide Plate (N=79)** | **Control (N=77)** | **P value** |
| Mechanical axis deviation (MAD) (mm) | -28.81 (4.10) | -27.96 (4.74) | 0.233 | -28.81 (4.10) | -27.97 (4.74) | 0.240 | -28.81 (4.10) | -27.97 (4.74) | 0.240 |
| ROM of knee flexion to extension (°) | 9.68 (10.66) | 3.70 (11.37) | 0.001 | 14.56 (11.01) | 11.17 (13.28) | 0.084 | 16.65 (12.50) | 16.36 (12.48) | 0.888 |
| Isometric Knee Flexion Strength (%of unaffected side) | 10.23 (2.16) | 9.82 (1.73) | 0.194 | 15.24 (2.19) | 14.91 (1.81) | 0.304 | 13.28 (2.26) | 13.21 (2.59) | 0.856 |
| Dynamic Knee Flexion Strength (%of unaffected side) | 10.01 (1.90) | 9.68 (2.19) | 0.306 | 13.08 (2.00) | 12.66 (2.23) | 0.225 | 11.04 (2.05) | 10.74 (2.56) | 0.423 |
| 30-s chair sit-to-stand test (times) | 2.92 (0.50) | 1.38 (1.80) | 0.000 | 3.91 (0.74) | 2.32 (1.74) | 0.000 | 2.89 (1.32) | 2.32 (1.83) | 0.029 |
| WOMAC-pain | -24.43 (3.40) | -21.04 (4.47) | 0.000 | -39.24 (4.32) | -39.61 (5.37) | 0.636 | -41.46 (4.95) | -41.69 (6.82) | 0.807 |
| WOMAC-function | -22.05 (3.15) | -18.68 (4.40) | 0.000 | -39.57 (3.17) | -39.26 (4.41) | 0.614 | -41.14 (3.53) | -40.84 (4.14) | 0.632 |
| WOMAC-Stiffness | -19.47 (6.25) | -14.27 (4.91) | 0.000 | -31.80 (6.27) | -31.52 (6.97) | 0.794 | -38.13 (9.16) | -38.16 (8.59) | 0.984 |
| Lysholm Knee Score | 9.89 (1.09) | 7.94 (1.16) | 0.000 | 13.90 (1.36) | 12.96 (1.21) | 0.000 | 15.87 (1.30) | 16.13 (1.25) | 0.212 |
| EQ-5D-5L-utility | 0.06 (0.08) | 0.06 (0.06) | 0.751 | 0.20 (0.10) | 0.21 (0.11) | 0.372 | 0.33 (0.11) | 0.33 (0.11) | 0.993 |

PP: per-protocol; ROM: range of motion; PROMs: patient-reported outcome measures; WOMAC: Western Ontario and McMaster Universities Osteoarthritis Index; EuroQol-5 Dimension, 5-Level.

Values represent the mean change from baseline for each group, reported as mean (standard deviation), with P values comparing between-group differences at each follow-up point. All outcome measures were unadjusted.

Supplementary Table 3 Effectiveness estimates from linear mixed effects models of the guiding plate group and the control group at month 3, 6 and 12 (per-protocol analysis)

| **PP^*^** | **3-month** | | | **6-month** | | | **12-month** | | |
| --- | --- | --- | --- | --- | --- | --- | --- | --- | --- |
|  | **Coefficient** | **95% CI** | **P value** | **Coefficient** | **95% CI** | **P value** | **Coefficient** | **95% CI** | **P value** |
| Mechanical axis deviation (MAD) (mm) | -0.482 | (-1.135, 0.171) | 0.148 | -0.614 | (-1.118, -0.110) | 0.017 | -0.679 | (-1.123, -0.236) | 0.003 |
| ROM of knee flexion to extension (°) | 2.961 | (1.392, 4.531) | 0.000 | 3.124 | (1.398, 4.850) | 0.000 | 2.368 | (0.645, 4.090) | 0.007 |
| Isometric Knee Flexion Strength (%of unaffected side) | 0.307 | (-1.193, 1.808) | 0.688 | 0.357 | (-1.152, 1.867) | 0.643 | 0.315 | (-1.179, 1.809) | 0.680 |
| Dynamic Knee Flexion Strength (%of unaffected side) | -0.116 | (-1.578, 1.346) | 0.876 | -0.038 | (-1.504, 1.428) | 0.959 | -0.030 | (-1.466, 1.407) | 0.968 |
| 30-s chair sit-to-stand test (times) | 0.600 | (0.182, 1.018) | 0.005 | 0.865 | (0.452, 1.279) | 0.000 | 0.732 | (0.318, 1.146) | 0.001 |
| WOMAC-pain | -1.795 | (-4.184, 0.594) | 0.141 | -0.843 | (-3.071, 1.384) | 0.458 | -0.591 | (-2.758, 1.576) | 0.593 |
| WOMAC-function | -1.643 | (-2.579, -0.706) | 0.001 | -0.994 | (-2.033, 0.046) | 0.061 | -0.792 | (-1.815, 0.231) | 0.129 |
| WOMAC-Stiffness | -2.689 | (-4.798, -0.579) | 0.012 | -1.817 | (-4.027, 0.394) | 0.107 | -1.387 | (-3.571, 0.798) | 0.213 |
| Lysholm Knee Score | 0.992 | (-0.630, 2.613) | 0.231 | 0.979 | (-0.616, 2.573) | 0.229 | 0.675 | (-0.892, 2.243) | 0.399 |
| EQ-5D-5L-utility | 0.000 | (-0.024, 0.025) | 0.990 | -0.007 | (-0.033, 0.019) | 0.599 | -0.006 | (-0.033, 0.021) | 0.642 |

PP: per-protocol; ROM: range of motion; PROMs: patient-reported outcome measures; WOMAC: Western Ontario and McMaster Universities Osteoarthritis Index; EuroQol-5 Dimension, 5-Level.

*Each coefficient represents the estimated between-group difference in the change from baseline (Guide Plate group minus Control group) for the specified outcome at that follow-up time point. Positive coefficients indicate higher scores in the Guide Plate group compared to the Control group, whereas negative values indicate lower scores in the Guide Plate group. Each estimate is presented with its 95% confidence interval (CI) and corresponding P value. All outcome measures were adjusted for baseline values in the model.

Supplementary Table 4 Costs of guide plate group and the control group at month 12 (per-protocol analysis)

| **PP** | **Guide Plate (N=79)** | **Control (N=77)** | **P value** |
| --- | --- | --- | --- |
| **Direct medical cost** (CNY) |  |  |  |
| Hospital stays cost (CNY) | 7014 (1026.69) | 6991 (1027.91) | 0.889 |
| Primary care cost (CNY) | 2435 (526.97) | 2470 (560.79) | 0.691 |
| Secondary care cost (CNY) | 3860 (3303.23) | 3835 (3232.04) | 0.962 |
| Physical therapist cost (CNY) | 13573 (1964.61) | 13229 (2003.44) | 0.280 |
| Implantation/Device/Medication (CNY) | 30163 (5046.43) | 24871 (4526.97) | 0.000 |
| **Direct non-medical cost** (CNY) |  |  |  |
| Transportation cost (CNY) | 1968 (281.35) | 1953 (282.91) | 0.742 |
| Nutrition cost (CNY) | 4521 (463.73) | 4553 (526.30) | 0.685 |
| **Opportunity Cost** (CNY) |  |  |  |
| Lost wages for patients (CNY) | 33592 (16310.94) | 33614 (14340.66) | 0.993 |
| Lost wages for families (CNY) | 3185 (4338.50) | 3213 (3835.64) | 0.966 |
| **Total cost** (CNY) | 100311 (17583.89) | 94729 (15449.35) | 0.037 |

PP: per-protocol; CNY: Chinese Yuan.

All relevant costs were captured from a societal perspective. This included direct medical costs (Hospital stay, primary and secondary care, rehabilitation and physical therapy costs and medication costs), direct non-medical costs related to care (transportation for medical visits and any specialized nutritional support during recovery), and indirect costs due to productivity loss (lost wages for patients during recovery or disability, and lost income for family members/caregivers, if applicable). Costs were obtained from hospital billing records and patient self-reports where needed (wage losses). All costs are reported in 2024 Chinese Yuan, as 2024 was the end of the enrollment period; costs incurred in earlier years were adjusted to 2024 price levels using the consumer price index. Costs and outcomes occurring beyond the initial year were discounted at an annual rate of 3% to reflect time preference, consistent with standard practice in health economic evaluations.

Supplementary Table 5 Adverse events and serious adverse events (intention-to-treat population)

| **AE** | **Guide Plate (N=90)** | **Control (N=90)** |
| --- | --- | --- |
| Patients with adverse events (no. [%]) ^#^ | 18 (20) | 20 (22) |
| Events related to surgery (no.) |  |  |
| Iatrogenic fracture of Lateral tibial cortex chain | 1 | 2 |
| Superficial infection treated with conservative method | 2 | 2 |
| Events related to assigned rehabilitation program (no.) |  |  |
| Pain | 4 | 5 |
| Swelling | 5 | 5 |
| Muscle strain | 2 | 2 |
| Knee arthromeningitis | 3 | 2 |
| Nausea and dizziness | 1 | 0 |
| Low back pain | 0 | 2 |
| Events unrelated to study (no.) |  |  |
| Ankle pain | 1 | 0 |
| Anxiety | 2 | 3 |
| **SAE*** |  |  |
| Patients with serious adverse events (no. [%]) | 4 (4.4) | 3 (3.3) |
| Events related to surgery (no.) |  |  |
| Deep infection required debridement | 1 | 0 |
| Nonunion required secondary bone graft | 0 | 1 |
| Events related to assigned rehabilitation program |  |  |
| Fall during exercise which result in waist fracture | 1 | 0 |
| Events unrelated to study (no.) |  |  |
| Hip fracture due to fall | 0 | 1 |
| Unplanned surgery | 2 | 1 |

AE: adverse events; SAE: serious adverse events.

*Patients with serious adverse events were automatically withdrawn from the study

#Some patients reported more than one adverse event.

Values of AEs and SAEs were reported as number (percentage) in intent-to-treat population

This study defines an Adverse Event (AE) as any unfavorable medical occurrence in a patient, regardless of causality, temporally associated with the 3D-printed guide or HTO surgery. A Serious Adverse Event (SAE) is an AE that results in death, is life-threatening, requires hospitalization, causes significant disability, or is a medically important event. All AEs and SAEs will be systematically collected from the time of informed consent through each of the follow-up time points (3-,6-,12-month). For each event, investigators will document a detailed description, date of onset and resolution, severity, required treatment, outcome, and an assessment of causality (related or not related to the device/procedure). All SAEs and any unanticipated device deficiencies must be reported to the coordinator of the study within 24 hours for expedited regulatory reporting, while all other AEs will be comprehensively recorded in the case report forms and summarized in the final study report.
